# Comparative efficacy and safety of infliximab and vedolizumab therapy in patients with inflammatory bowel disease: a systematic review and meta-analysis

**DOI:** 10.1186/s12876-022-02347-1

**Published:** 2022-06-08

**Authors:** Laurent Peyrin-Biroulet, Perttu Arkkila, Alessandro Armuzzi, Silvio Danese, Jordi Guardiola, Jørgen Jahnsen, Charles Lees, Edouard Louis, Milan Lukáš, Walter Reinisch, Xavier Roblin, Minyoung Jang, Han Geul Byun, Dong-Hyeon Kim, Sung Jeong Lee, Raja Atreya

**Affiliations:** 1 Centre Hospitalier Régional Universitaire de Nancy, Nancy, France; 2 Department of Gastroenterology, Helsinki University and Helsinki University Hospital, Helsinki, Finland; 3 Humanitas Research Hospital, Milan, Rozzano Italy; 4 Gastroenterology and Endoscopy, University Vita-Salute San Raffaele, Milan, Italy; 5 Digestive Diseases Department, Bellvitge University Hospital, Bellvitge Biomedical Research Institute-IDIBELL, University of Barcelona, L’Hospitalet de Llobregat, Barcelona, Spain; 6 Department of Gastroenterology, Institute of Clinical Medicine, Akershus University Hospital, University of Oslo, Oslo, Norway; 7 Center of Genomics and Experimental Medicine, University of Edinburgh, Edinburgh, UK; 8 Department of Gastroenterology, University Hospital CHU of Liège, Liège, Belgium; 9 ISCARE Clinical Centre, Prague, Czech Republic; 10 Medical University of Vienna, Vienna, Austria; 11 University Hospital of Saint-Etienne, Saint-Etienne, France; 12 Celltrion Healthcare, Incheon, Republic of Korea; 13 Medical Department 1, University Hospital Erlangen, Friedrich-Alexander-University of Erlangen-Nürnberg, Ulmenweg 18, 91054 Erlangen, Germany

**Keywords:** Biological therapy, Tumor Necrosis Factor-alpha, Antibodies, Monoclonal, IBD, Crohn’s disease, Ulcerative colitis

## Abstract

**Background and aims:**

There are limited comparative data for infliximab and vedolizumab in inflammatory bowel disease patients.

**Methods:**

We conducted a systematic review and meta-analysis to compare the efficacy and safety of infliximab and vedolizumab in adult patients with moderate-to-severe Crohn’s disease or ulcerative colitis.

**Results:**

We identified six eligible Crohn’s disease and seven eligible ulcerative colitis trials that randomised over 1900 participants per disease cohort to infliximab or vedolizumab. In the Crohn’s disease and ulcerative colitis cohorts, infliximab yielded better efficacy than vedolizumab for all analysed outcomes (CDAI-70, CDAI-100 responses, and clinical remission for Crohn’s disease and clinical response and clinical remission for ulcerative colitis) during the induction phase, with non-overlapping 95% confidence intervals. In the maintenance phase, similar proportions of infliximab- or vedolizumab-treated patients achieved clinical response, clinical remission, or mucosal healing in both Crohn’s disease and ulcerative colitis. For the safety outcomes, rates of adverse events, serious adverse events, and discontinuations due to adverse events were similar in infliximab- and vedolizumab-treated patients in both diseases. The infection rate was higher in infliximab for Crohn’s disease and higher in vedolizumab when treating patients with ulcerative colitis. There was no difference between the treatments in the proportions of patients who reported serious infections in both indications.

**Conclusions:**

Indirect comparison of infliximab and vedolizumab trials in adult patients with moderate-to severe Crohn’s disease or ulcerative colitis demonstrated that infliximab has better efficacy in the induction phase and comparable efficacy during the maintenance phase and overall safety profile compared to vedolizumab.

**Supplementary Information:**

The online version contains supplementary material available at 10.1186/s12876-022-02347-1.

## Introduction

Inflammatory bowel diseases (IBD) is a heterogeneous group of chronic inflammatory disorders that mainly affects the gastrointestinal tract, of which the principal phenotypes are Crohn’s disease (CD) [1] and ulcerative colitis (UC) [2]. Several biological treatment options are available. Tumour necrosis factor-α inhibitors (TNFis), such as infliximab and adalimumab, were the first class of biological agents approved for the treatment of patients with IBD and are highly effective against luminal and extra-intestinal manifestations of the disease [3–9]. Anti-integrin agents (e.g., vedolizumab and natalizumab—only in the United States) are the second class of biological agents that have proven effective in both IBD entities.

Treatment guidelines for CD recommend TNFis for patients who have not responded to conventional therapy (e.g., steroids and/or thiopurines), whereas vedolizumab and ustekinumab, anti-interleukin (IL)-12 and IL-23, are recommended for patients who have had an inadequate response to conventional therapy and/or TNFis [10]. The use of TNFi therapy early in the disease course (in the first 2 years) may be more effective in CD and could be particularly beneficial in patients with poor prognostic factors (e.g., in patients with fistulising perianal disease) [10]. Guidelines for UC recommend treatment escalation with thiopurines, TNFi therapy, vedolizumab, ustekinumab or tofacitinib for patients receiving high dose mesalazine maintenance therapy who become corticosteroid dependent or refractory [11]. In the case of TNFi treatment failure, second-line therapy with vedolizumab, ustekinumab or tofacitinib should be considered [11]. US guidelines are broadly aligned with European guidelines with respect to appropriate biological therapies for patients with moderate-to-severe IBD [12, 13].

Guidelines recommend that the choice of first-line biological agent should be determined by clinical factors, cost, safety, availability of local infusion capacity, as well as patient preference and likely adherence [11]; however, there is limited evidence regarding the comparative efficacy and safety of these agents for the treatment of these patient populations. No head-to-head RCTs have compared infliximab and vedolizumab for the treatment of patients with IBD, and comparative data from real-world studies [14, 15] are difficult to contextualise in the absence of mutually supplementary RCTs [16].

Several systematic reviews have synthesised data for multiple biological agents (including infliximab and vedolizumab) in IBD to draw preliminary conclusions; however, one did not evaluate the relative safety of infliximab and vedolizumab [17], and the other covered only the induction phase in patients with UC [18]. Furthermore, existing systematic reviews do not include data from more recent pivotal trials (e.g., of subcutaneous [SC] infliximab) [19]. Therefore, to our knowledge, we have conducted the first systematic review and meta-analysis to comprehensively evaluate the comparative efficacy and safety of infliximab and vedolizumab in adult patients with moderate-to-severe CD or UC.

## Methods

The current systematic review was performed using a pre-established protocol. (PROSPERO number: CRD42021177954) [20].

### Search strategy

We performed systematic electronic searches of PubMed, Embase and the Cochrane Library (comprising the Cochrane Database of Systematic Reviews, Database of Abstracts of Reviews of Effects, Cochrane Central Register of Controlled Trials, and the Health Technology Assessment database). Search strategies were developed using Medical Subject Headings and free-text terms (Supplementary materials). All searches were performed for the period of 1 January 2010 through 30 April 2021 to ensure the inclusion of recently published data.

### Criteria for considering studies for this review

#### Study design

Parallel-group RCTs were included for the analysis.

#### Participants

Two cohorts of patients (analysed separately) were included: adults (aged ≥ 18 years) with moderate-to-severe CD or adults (aged ≥ 18 years) with moderate-to-severe UC. Patients with unspecified disease severity or those who had undergone intestinal surgery were excluded.

#### Interventions

We included trials that evaluated infliximab (reference product or biosimilar) or vedolizumab. Dosing regimens were required to align with the summary of product characteristics (SmPC) for approved drugs, or with the SmPC of the originator product for unapproved biosimilars.

#### Outcomes

Studies that reported one or more of the following outcomes at Week 6 (induction phase) and/or at 1 year (Weeks 50–54; maintenance phase) were included. Efficacy outcomes for CD included the proportion of patients achieving a Crohn’s Disease Activity Index (CDAI)-70 response, defined as a 70 ≥ points decrease from the baseline value, proportion of patients achieving a CDAI-100 response (a decrease in CDAI score of ≥ 100 points from the baseline value) and proportion of patients achieving clinical remission (an absolute CDAI score of < 150 points). Efficacy outcomes for UC included the proportion of patients achieving clinical response (defined as a decrease from baseline in total Mayo score of ≥ 3 points and ≥ 30%, with an accompanying decrease in rectal bleeding subscore of ≥ 1 point or an absolute rectal bleeding subscore of 0 or 1), proportion of patients achieving clinical remission (a total Mayo score of ≤ 2 points with no individual subscore exceeding 1 point) and proportion of patients achieving mucosal healing (an absolute endoscopic subscore of 0 or 1 per the Mayo Scoring System). Safety outcomes (CD and UC) included the proportions of patients experiencing any adverse event (AE), serious adverse event (SAE), any infection or serious infection, and the proportion who discontinued due to AEs or lack of efficacy that are evaluated at any point of time in a year.

### Study selection

Two investigators (HGB, MJ) independently screened the titles and abstracts of the retrieved records (per eligibility criteria in Sect. 2.2) to exclude studies that are irrelevant to the research question. A third reviewer (Taek Sang Kwon, Celltrion Healthcare) mediated in the points of disagreement. The third reviewer randomly selected sample of excluded studies to validate appropriate application of the exclusion criteria.

Full-text articles of studies identified as potentially relevant for inclusion during title and abstract screening were reviewed independently by two authors HGB and MJ to determine inclusion (recording reasons), and the third reviewer arbitrated in the case of disagreement. Multiple reports of the same study were collected so that studies were the unit of interest for the review. The screening and full-text review process was thoroughly documented to complete a Preferred Reporting Items for Systematic Reviews and Meta-Analyses (PRISMA) flow diagram [21].

### Data extraction and management

Study characteristics and outcome data were extracted from the included studies and recorded using a Microsoft Excel template (Microsoft Corp., Redmond, WA, USA). The following study characteristics were extracted: design (study duration, randomisation method, blinding), population (demographics, baseline disease activity, number of randomised participants, prior TNFi use, concomitant medication), interventions (type, dose, regimen), and prespecified outcome measures (see above 2.2.4; Additional file 1: Table 1).

### Data synthesis and measures of treatment effect

Data for each prespecified outcome of interest were pooled in two separate analyses for patients with CD or UC, respectively. Outcomes reported as proportions (*n:* event*; N:* population) were analysed, and the overall proportions from each study were combined using a random-effects meta-analysis. A meta-analysis was only performed if studies were deemed to have similar characteristics (e.g., study populations and treatments). The *I*^2^ statistic was used to evaluate heterogeneity among the trials included in each meta-analysis. All statistical analyses were performed using R (version 4.0.2).

### Quality assessment

Risk of bias and generalisability for the included studies were evaluated according to criteria defined in the *Cochrane Handbook for Systematic Reviews of Interventions* [22]. The following domains were utilised in order to assess the risk of bias: random sequence generation; allocation concealment; blinding of participants and personnel, blinding of outcome assessment; incomplete outcome data; selective outcome reporting; and other bias. Each potential source of bias was rated as high, low, or unclear. Assessments were completed by an author (HGB) responsible for data extraction and checked by a second author (MJ).

## Results

### Search results

**Fig. 1:**
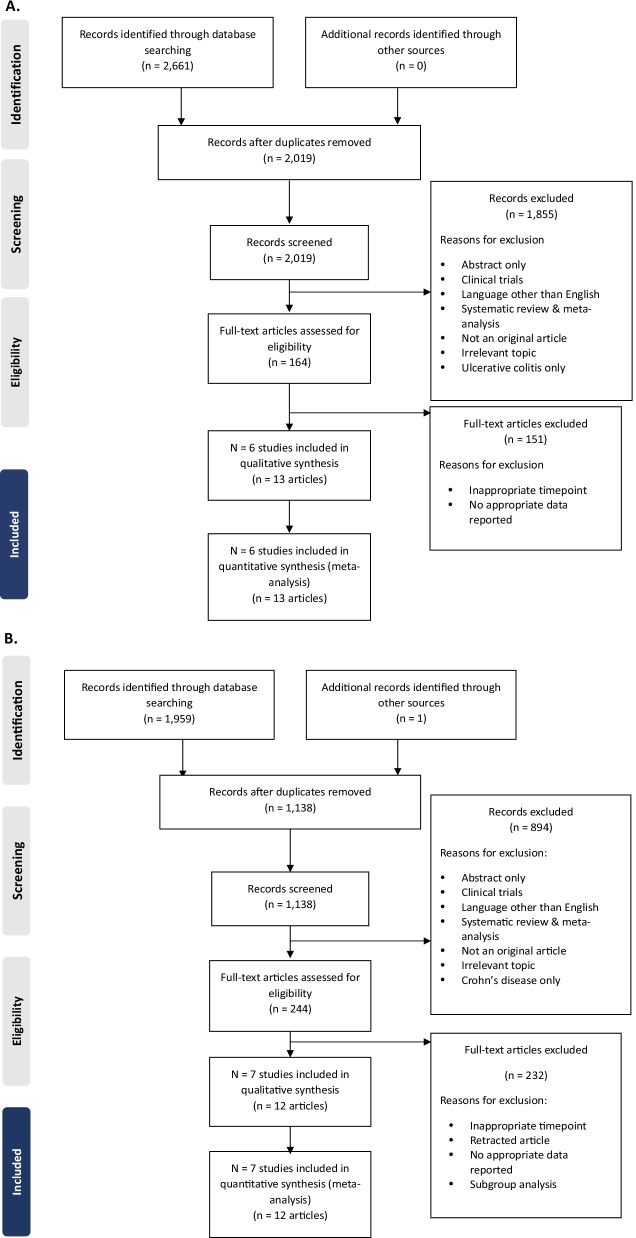
PRISMA flow diagrams for **A** Crohn’s disease and **B** ulcerative colitis. Abbreviation: *PRISMA* Preferred reporting items for systematic reviews and meta-analyses

The selection of studies for inclusion is summarised in a PRISMA flow diagram to illustrate the flow of information for studies enrolling patients with CD (Fig. 1A). We identified 2,661 records through the searches. After removal of duplicates, 2,019 records were screened (1,855 records excluded) and 164 full-text articles were assessed against the eligibility criteria (150 articles excluded). Six studies (reported in 13 articles) were included in the qualitative synthesis and in the quantitative synthesis, as follows:

- Infliximab (four studies): NCT00094458 (SONIC) [23–26], NCT02096861 (PLANET CD) [6, 27], NCT02148640 (NOR-SWITCH) [28, 29], NCT02883452 (CT-P13 SC trial) [19].
- Vedolizumab (two studies): NCT00783692 (GEMINI 2) [30–33], NCT01224171 (GEMINI 3) [31, 32, 34].

**Fig. 2:**
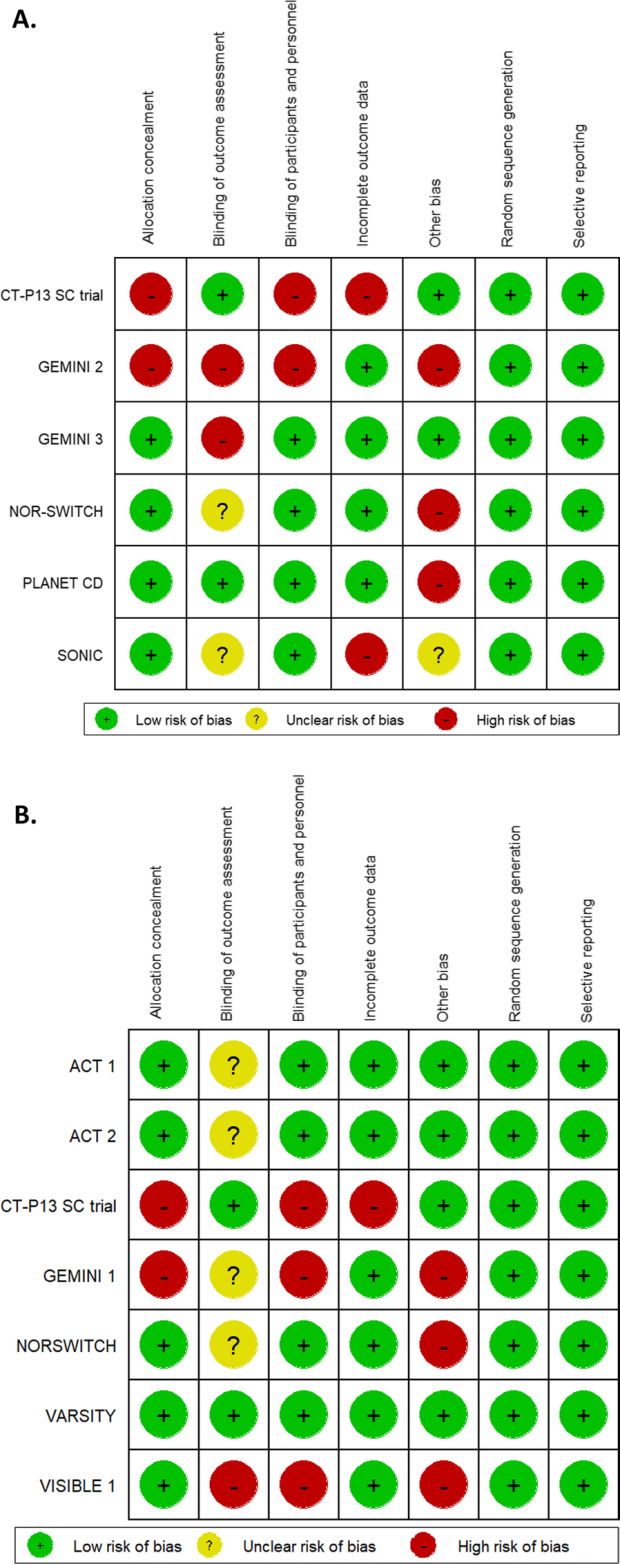
Quality assessment results for studies contributing data to the analyses for **A** Crohn’s disease and **B** ulcerative colitis. Panel **A**: Risk of bias determined based on assessment of the following study publications: SONIC, NOR-SWITCH, GEMINI 2, GEMINI 3, CT-P13 SC trial, and PLANET CD. Abbreviation: SC, subcutaneous. Panel **B**: Risk of bias determined based on assessment of the following study publications: GEMINI 1, NOR- SWITCH, ACT 1, ACT 2, VISIBLE 1, VARSITY, and CT-P13 SC trial. Abbreviation: SC, subcutaneous

A PRISMA flow diagram summarising the flow of information for studies enrolling patients with UC is presented in Fig. 1B. We identified 1,959 records through the electronic searches and one record from another source. Specifically, as data for ACT 1 and 2 study by Adedokun et al. (2018) was not utilizable in our analysis because the article did not evaluate the prespecified timeframe and outcomes of our interest [35]. The current review evaluated ACT 1 and 2 data as more data were needed for UC in order to perform meta-analyses in some outcomes (Fig. 2A, Additional file 1: Figs. 11, 12, 18), largely accounting for the fact that infliximab had few available RCT data for the past decade. Thus, we instead have taken data from the reference list of the article as an exception [5]. After removal of duplicates, 1,138 records were screened (894 articles excluded) and 244 full-text articles were assessed against the eligibility criteria (232 articles excluded). Seven studies (reported in 12 articles) were included in the qualitative synthesis and quantitative analyses, as follows:

- Infliximab (four studies): NCT00036439 (ACT1) [5, 35], NCT00096655 (ACT2) [5, 35], NCT02148640 (NOR-SWITCH) [28, 29], NCT02883452 (CT-P13 SC trial) [19].
- Vedolizumab (three studies): NCT00783718 (GEMINI 1) [36–40], NCT02497469 (VARSITY) [41], NCT02611830 (VISIBLE 1) [42].

### Study characteristics

#### Studies contributing to CD analyses

The design and eligibility criteria of the six studies that contributed data to the CD analyses were generally consistent (Additional file 1: Table 1). All six studies were randomised trials with a duration of ≥ 50 weeks: five studies included a double-blind period, and one study was conducted using an open-label design (CT-P13 SC trial). Two of the six studies included an open-label extension (NOR-SWITCH and GEMINI 3) and three studies (PLANET CD, NOR-SWITCH and CT-P13) included switching phases wherein participants switched between infliximab products. Five of six studies were multinational, whereas one study was conducted in Norway (NOR-SWITCH).

Across studies, inclusion criteria required participants to be adults (aged ≥ 18) with a diagnosis of CD; four of six studies required participants to have a CDAI score of 220–450, one study (GEMINI 3) specified 220–400 and another (NOR-SWITCH) did not specify a CDAI score. Prior TNFi use was not permitted in three studies (SONIC, PLANET CD, CT-P13 SC trial), stable treatment with infliximab for ≥ 6 months was an inclusion criterion in NOR-SWITCH, and treatment failure with corticosteroids, immunosuppressive agents or TNFis was an inclusion criterion for GEMINI 2 and GEMINI 3 (within the past 5 years).

All studies included a treatment arm of either infliximab or vedolizumab. An intravenous formulation of infliximab or vedolizumab was initially administered at Weeks 0, 2, and 6 for induction and every 8 weeks (Q8W) thereafter while a subcutaneous formulation of infliximab was initially administered at Weeks 0 and 2 for induction and every 2 weeks (Q2W) from Week 6 [43, 44].

A total of 2,020 participants were initially randomised/assigned to relevant treatment arms of the selected studies. The mean/median age ranged from 32.0 to 39.5 years, 39% to 56% of participants were female, mean/median body weight ranged from 66.1 to 72.0 kg (where reported) and mean/median disease duration ranged from 2.2 to 14.3 years (Additional file 1: Table 2).

#### Studies contributing to UC analyses

The design and eligibility criteria of the seven studies that contributed data to the UC analyses were generally consistent (Additional file 1: Table 1). All seven studies were randomised trials with a duration of ≥ 46 weeks, except for ACT2 (22 weeks): six studies included double-blind periods and one study was open-label (CT-P13 SC trial). Two studies included an open-label extension (NOR-SWITCH and VISIBLE 1). Three studies included switching phases wherein participants switched between infliximab products (NOR-SWITCH and CT-P13 SC trial) or between IV and SC vedolizumab (VISIBLE 1). Six of the seven studies were multinational, whereas one study (NOR-SWITCH) was conducted in Norway.

Across studies, inclusion criteria required participants to be adults (aged ≥ 18 years) with active UC (Mayo score 6–12 and endoscopic sub-score ≥ 2) despite treatment with conventional therapies (e.g., corticosteroids, azathioprine or mercaptopurine). Prior TNFi treatment was not permitted in four studies (ACT 1, ACT 2, CT-P13 SC trial and VARSITY), stable treatment with infliximab for ≥ 6 months was an inclusion criterion in NOR-SWITCH and use of TNFi and biological agents were not permitted within 60 days before study initiation in GEMINI 1 and VISIBLE 1, respectively.

Four of the seven studies evaluated infliximab (ACT 1, ACT 2, NOR-SWITCH and CT-P13 SC trial) and three evaluated vedolizumab (GEMINI 1, VISIBLE 1 and VARSITY). All of the infliximab studies included a treatment arm wherein infliximab was administered at Weeks 0, 2 and 6 (induction) and Q8W thereafter (maintenance) except for the CT-P13 SC trial. The CT-P13 SC trial included evaluation of subcutaneous infliximab Q2W from Week 6 following IV induction at Weeks 0 and 2 (see Sect. 3.2.1). All of the vedolizumab studies included a treatment arm wherein IV vedolizumab was administered at Weeks 0, 2, and 6 and Q8W thereafter except for one study that included a treatment arm wherein IV vedolizumab was administered at Weeks 0 and 2 for induction, followed by SC vedolizumab Q2W from Week 6.

A total of 1,999 participants were initially randomised to relevant treatment arms of the included studies. The mean/median age ranged from 33.0 to 45.8 years, 30% to 46% of participants were female, mean/median body weight ranged from 66.1 to 80.0 kg (where reported) and mean/median disease duration ranged from 5.7 to 11.5 years (Additional file 1: Table 3).

### Risk of bias and generalisability in the included studies

**Fig. 3:**
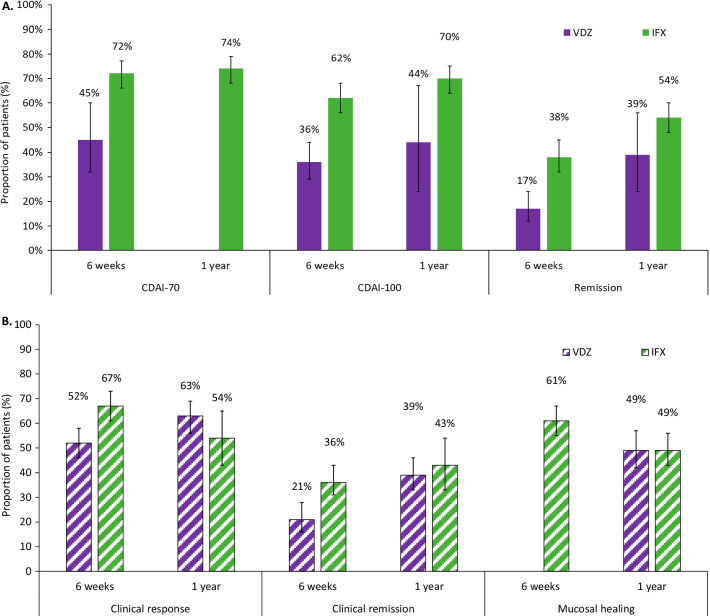
**A** A comparison of infliximab versus vedolizumab for key efficacy outcomes in patients with Crohn’s disease. Abbreviation: CDAI, Crohn’s Disease Activity Index; IFX, infliximab; VDZ, vedolizumab. **B** A comparison of infliximab versus vedolizumab for key efficacy outcomes in patients with ulcerative colitis. Abbreviation: IFX, infliximab; VDZ, vedolizumab

A summary of the risk-of-bias assessment for studies contributing to the CD analyses is presented in Fig. 3A. Across 42 assessments (six studies and seven risk-of-bias domains), 29 were considered to be at low risk of bias, 10 at high risk and three to have an unclear risk of bias. All six studies had low risk of bias for random sequence generation and selective reporting. NOR-SWITCH was the only study considered at low or unclear risk of bias for all domains. Most studies (five out of six) had one or more domains considered to be at high risk, and the CT-P13 SC and GEMINI 2 trials were at high risk of bias for three and four domains, respectively. The CT-P13 SC study was considered to be at high risk of bias due to the nature of an open-label trial and the results were combined as inflammatory bowel disease, not categorized into CD and UC. GEMINI 2, NOR-SWITCH, and PLANET CD were considered to be at high risk of ‘other bias’ because of the selective inclusion of induction responders in the maintenance phase; notably, this high risk of bias applies only to the maintenance-phase data for these studies as the induction phase included all enrolled patients.

Figure 3B presents a summary of the risk-of-bias assessment for studies contributing to the UC analyses. Across 49 assessments (seven studies and seven risk-of-bias domains), 36 were considered to be at low risk of bias, nine at high risk and four to have an unclear risk of bias. All seven studies were considered to be at low risk of bias for random sequence generation and selective reporting. Four studies were considered at low or unclear risk of bias for all domains (VARSITY, ACT 1, ACT 2, NOR-SWITCH). Three studies had three domains considered to be at high risk of bias (GEMINI 1, VISIBLE 1, CT-P13 SC trial). The results from CT-P13 SC trial have the same risk of bias as CD such as having an open-label study design and did not fully categorised the safety outcomes. GEMINI 1 and VISIBLE 1 were considered to be at high risk of ‘other bias’ because of the selective inclusion of induction responders in the maintenance phase; again, this high risk of bias applies only to the maintenance-phase data for these studies as the induction phase included all enrolled patients.

### Comparative efficacy and safety between the treatments in treating IBD

**Fig. 4:**
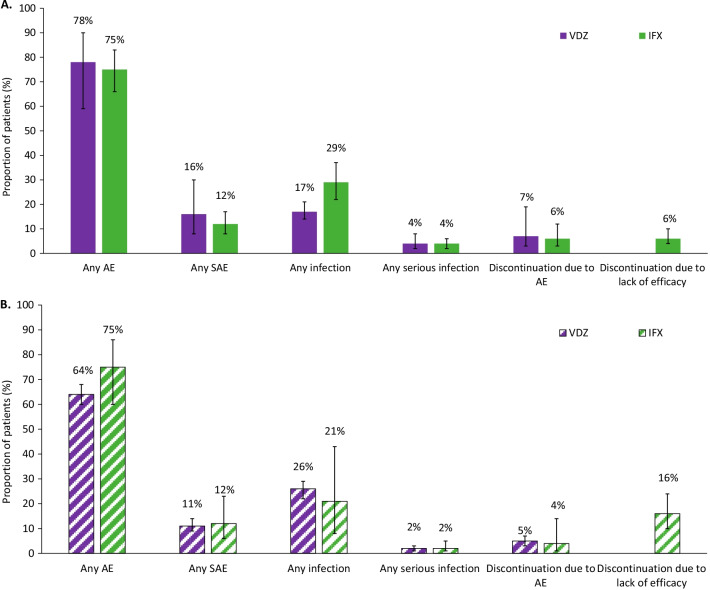
**A** A comparison of infliximab versus vedolizumab for key safety outcomes in patients with Crohn’s disease (≤ 1 year) Abbreviation: AE, adverse events; SAE, serious adverse events; IFX, infliximab; VDZ, vedolizumab. **B**. A comparison of infliximab versus vedolizumab for key safety outcomes in patients with ulcerative colitis (≤ 1 year). Abbreviation: AE, adverse events; SAE, serious adverse events; IFX, infliximab; VDZ, vedolizumab

**Fig. 5:**
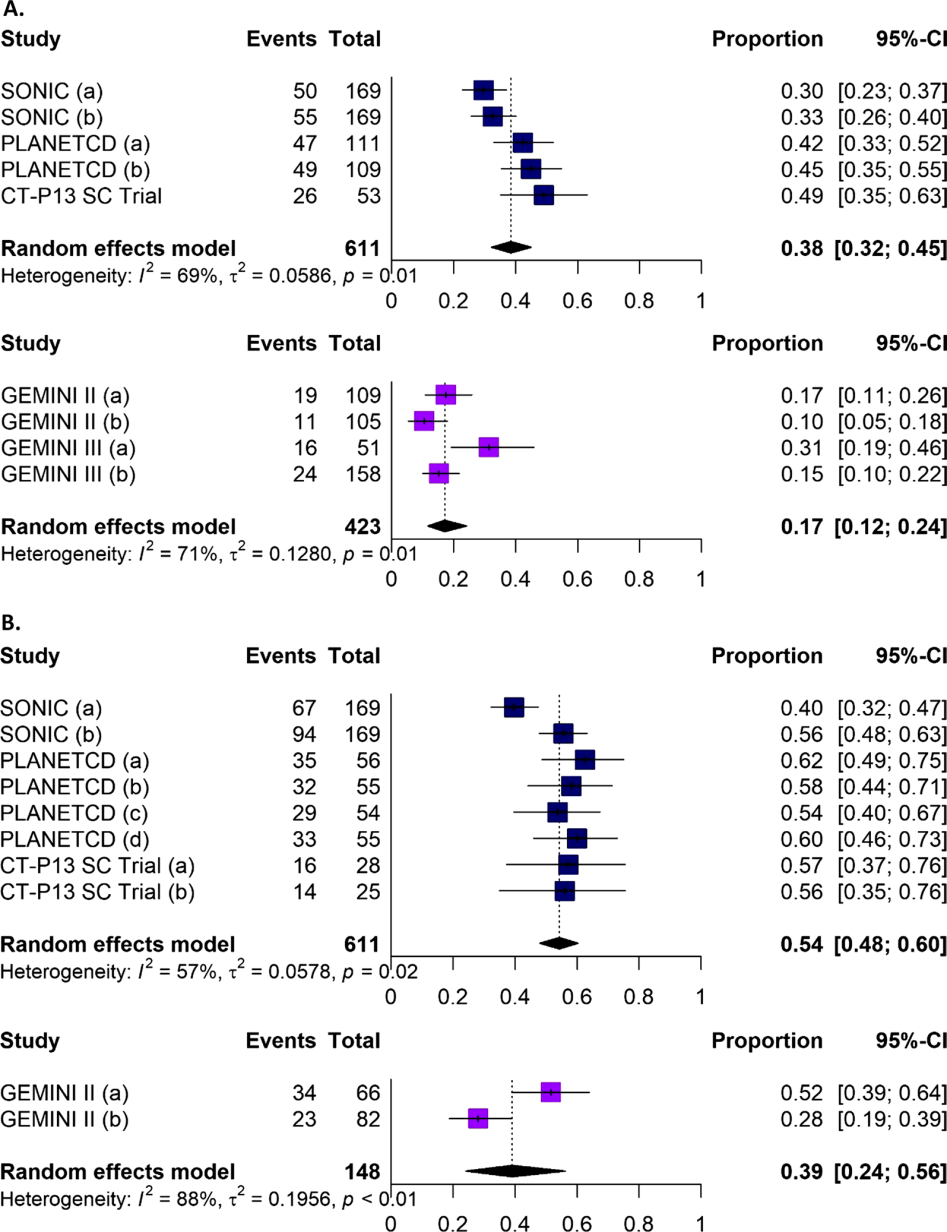
Forest plots showing the proportion of patients with Crohn’s disease achieving clinical remission during **A** the induction phase and **B** the maintenance phase with infliximab (upper plot) or vedolizumab (lower plot). Panel A SONIC (a): IFX IV (corticosteroid free); SONIC (b): combination therapy; PLANET CD (a): patients with CT-P13 IV only; PLANET CD (b): patients with CT-P13 IV and IFX IV; GEMINI 2 (a): VDZ before TNFi; GEMINI 2 (b): VDZ after TNFi failure; GEMINI 3 (a): VDZ IV before TNFi; GEMINI 3 (b): VDZ IV after TNFi failure. Abbreviation: CI, confidence interval; IFX, infliximab; IV, intravenous; TNFi, tumour necrosis factor-α inhibitor; VDZ, vedolizumab. Panel B SONIC (a): IFX IV (corticosteroid free); SONIC (b): combination therapy; PLANET CD (a): CT-P13 IV only; PLANET CD (b): CT-P13 IV switch to IFX IV; PLANET CD (c): IFX IV only; PLANET CD (d): IFX IV switch to CT-P13 IV; CT-P13 SC trial (a): CT-P13 SC only; CT-P13 SC trial (b): CT-P13 IV switch to CT-P13 SC; GEMINI 2 (a): VDZ before TNFi; GEMINI 2 (b): VDZ after TNFi failure. Abbreviation: CI, confidence interval; IFX, infliximab; IV, intravenous; SC, subcutaneous; TNFi, tumour necrosis factor-α inhibitor; VDZ, vedolizumab

**Fig. 6:**
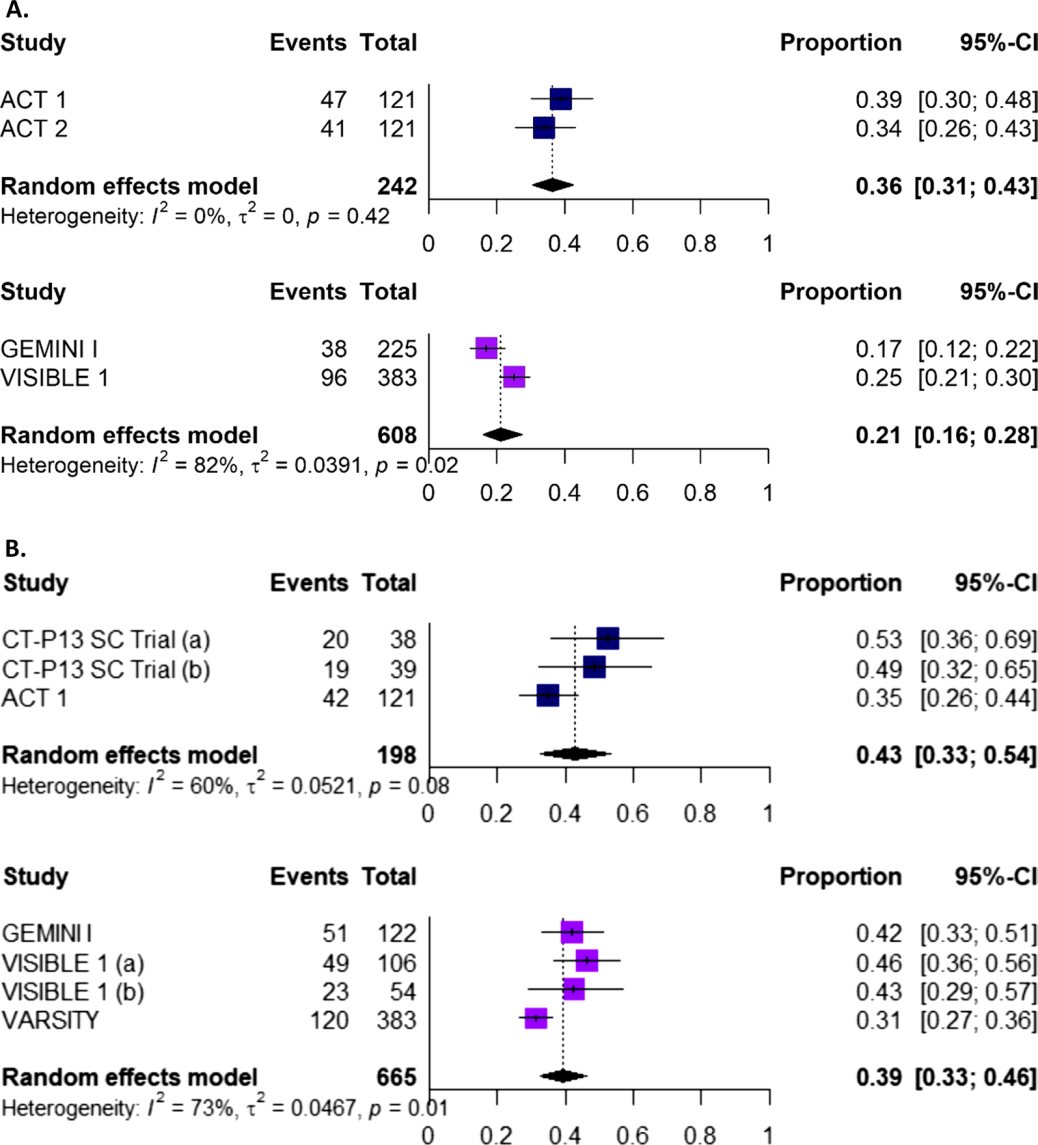
Forest plots showing the proportion of patients with ulcerative colitis achieving clinical remission during **A** the induction phase and **B** the maintenance phase with infliximab (upper plot) or vedolizumab (lower plot). Panel **A** Abbreviation: CI, confidence interval. Panel **B** CT-P13 SC trial (a): CT-P13 SC only; CT-P13 SC trial (b): CT-P13 IV switch to CT-P13 SC; VISIBLE 1 (a): VDZ SC; VISIBLE 1 (b): VDZ IV Abbreviation: CI, confidence interval; IV, intravenous; SC, subcutaneous

A summary of findings for the meta-analyses for infliximab and vedolizumab in patients with CD is presented in Table 1. For most efficacy outcomes during the induction and maintenance phases, infliximab yielded better efficacy than vedolizumab, with non-overlapping 95% confidence intervals (CIs) (Fig. 4A). During the induction phase, pooled results for efficacy outcomes in patients with CD showed that a higher proportion of patients treated with infliximab achieved a CDAI-70 response, CDAI-100 response or clinical remission with non-overlapping 95% CIs, in comparison with patients treated with vedolizumab (Fig. 5A, Additional file 1: Figs. 1–2). In the maintenance phase, a CDAI-70 response was not reported for vedolizumab, so only the data for infliximab is presented (Additional file 1: Fig. 3); a numerical advantage with overlapping 95% CIs was observed with infliximab over vedolizumab for CDAI-100 and clinical remission (Fig. 5B, Additional file 1: Figs. 4). Pooled results for safety outcomes (Fig. 6A; Additional file 1: Figs. 5–10) showed that the proportions of patients experiencing AEs, SAEs, or who discontinued due to AEs were similar in infliximab- and vedolizumab-treated patients. A higher rate of infection was reported with infliximab; however, when it comes to serious infections, similar rates between infliximab and vedolizumab are observed. Six percent of patients treated with infliximab discontinued because the treatment was ineffective (Additional file 1: Fig. 10) while one study was available for vedolizumab, where almost one-third of patients (37.7%) discontinued vedolizumab treatment due to lack of efficacy in the maintenance phase [30].

**Table 1:** Comparative efficacy and safety between infliximab and vedolizumab in patients with Crohn’s disease

Outcome	Treatment	Participants, *N*	Infliximab, estimate (95% CI)^a^	Heterogeneity (%)
Efficacy: Induction phase (Week 6)				
CDAI-70	Infliximab	220	72% (66%, 77%)	0
	Vedolizumab	214	45% (32%, 60%)	89
CDAI-100	Infliximab	273	62% (56%, 68%)	0
	Vedolizumab	423	36% (29%, 44%)	68
Clinical remission	Infliximab	611	38% (32%, 45%)	69
	Vedolizumab	423	17% (12%, 24%)	71
Efficacy: Maintenance phase (Week 50 to 54)				
CDAI-70	Infliximab	220	74% (68%, 79%)	0
CDAI-100	Infliximab	273	70% (64%, 75%)	0
	Vedolizumab	148	44% (24%, 67%)	93
Clinical remission	Infliximab	611	54% (48%, 60%)	57
	Vedolizumab	148	39% (24%, 56%)	88
Safety (≤ 1 year)				
Any AE	Infliximab	717	75% (66%, 83%)	86
	Vedolizumab	985	78% (59%, 90%)	98
Any SAE	Infliximab	717	12% (8%, 17%)	65
	Vedolizumab	993	16% (8%, 30%)	93
Any infection	Infliximab	717	29% (22%, 37%)	80
	Vedolizumab	427	17% (14%, 21%)	24
Any serious infection	Infliximab	562	4% (2%, 6%)	0
	Vedolizumab	985	4% (2%, 8%)	67
Discontinuation due to AE	Infliximab	717	6% (3%, 12%)	79
	Vedolizumab	976	7% (3%, 15%)	84
Discontinuation due to lack of efficacy	Infliximab	311	6% (4%, 10%)	49

^a^ Random-effects model

All results reported as the proportion of patients achieving the response

AE Adverse event, *CDAI* Crohn’s disease activity index, *CI* Confidence interval, *SAE* Serious adverse event

The findings for the meta-analyses for infliximab and vedolizumab in patients with UC are presented in Figs. 4B and 6B (Additional file 1: Figs. 11–20), with a summary presented in Table 2. Pooled results for efficacy outcomes in patients with UC showed that in the induction phase, a higher proportion of patients treated with infliximab achieved a clinical response or clinical remission with non-overlapping 95% CIs, compared with patients treated with vedolizumab (Fig. 2A, Additional file 1: Fig. 11). In the maintenance phase, similar proportions of patients treated with infliximab or vedolizumab achieved a clinical response, clinical remission or mucosal healing, with overlapping 95% CIs (Fig. 2B; Additional file 1: Figs. 13–14). Pooled results for safety outcomes showed that the proportions of patients experiencing AEs or infections, or who discontinued due to AEs, were similar in the infliximab and vedolizumab groups (Fig. 6B; Additional file 1: Figs. 15–20); rates of SAEs and serious infections were also similar with overlapping 95% CIs. Fourteen percent of patients with vedolizumab discontinued due to lack of efficacy (Additional file 1: Fig. 20); no available was available for infliximab.

**Table 2:** Comparative efficacy and safety between infliximab and vedolizumab in patients with ulcerative colitis

Outcome	Treatment	Participants, *N*	Infliximab and biosimilars, estimate (95% CI)^a^	Heterogeneity (%)
Efficacy: Induction phase (Week 6)				
Clinical response^b^	Infliximab	242	67% (61%, 73%)	0
	Vedolizumab	608	52% (46%, 58%)	78
Mucosal healing	Infliximab	242	61% (55%, 67%)	0
Clinical remission^b^	Infliximab	242	36% (31%, 43%)	0
	Vedolizumab	608	21% (16%, 28%)	82
Efficacy: Maintenance phase (Week 50 to 54)				
Clinical response^b^	Infliximab	198	54% (43%, 65%)	62
	Vedolizumab	282	63% (56%, 69%)	51
Mucosal healing	Infliximab	198	49% (43%, 56%)	2
	Vedolizumab	665	49% (42%, 57%)	78
Clinical remission^b^	Infliximab	198	43% (33%, 54%)	60
	Vedolizumab	665	39% (33%, 46%)	73
Safety (≤ 1 year)				
Any AE	Infliximab	214	75% (60%, 86%)	86
	Vedolizumab	543	64% (60%, 68%)	44
Any SAE	Infliximab	214	12% (6%, 23%)	72
	Vedolizumab	543	11% (9%, 14%)	0
Any infection	Infliximab	214	21% (8%, 43%)	91
	Vedolizumab	543	26% (22%, 29%)	14
Any serious infection	Infliximab	242	2% (1%, 5%)	0
	Vedolizumab	489	2% (1%, 3%)	0
Discontinuation due to AE	Infliximab	214	4% (1%, 14%)	0
	Vedolizumab	667	5% (3%, 7%)	0
Discontinuation due to lack of efficacy	Vedolizumab	667	15% (10%, 22%)	82

^a^ Random-effects model

^b^ Based on total Mayo score

All results reported as the proportion of patients achieving the response

AE Adverse event, *CDAI* Crohn’s disease activity index, CI Confidence interval, *SAE* Serious adverse event

## Discussion

The present study is the first systematic review to compare the efficacy and safety of infliximab and vedolizumab in adult patients with moderate-to-severe CD or moderate-to-severe UC in order to address a lack of evidence of a direct comparison between the treatments. Data were extracted and pooled for the prespecified outcomes of interest at the corresponding 6-week and/or 50- to 54-week timepoints, respectively. Notably, the present evidence synthesis is the first to our knowledge to incorporate data for CT-P13 SC, an SC formulation of the infliximab.

Our results show that infliximab yielded better efficacy than vedolizumab for all the efficacy outcomes in patients with CD or UC during the induction phase, and comparable clinical efficacies with overlapping 95% CI in both diseases during the maintenance phase. The safety profiles of infliximab and vedolizumab in both cohorts were generally similar in terms of the proportions of patients experiencing AEs, SAEs, infection, and serious infection, as well as the rates of discontinuations due to AEs in the analysed study period.

Based on the demographics and clinical characteristics of the study populations contributing to the review, the present findings are applicable to patients with moderate-to-severe CD or UC and support the use of infliximab as a first-line biologic in these populations, per guideline recommendations. The quality of the evidence was broadly considered to be moderate to high on the guidelines. Prespecified outcomes of interest were well reported in the included studies, and meta-analyses included ≥ 200 patients for the majority of outcomes evaluated.

Risk of bias in the included studies was principally considered to be low or was unclear (i.e. due to a lack of necessary information in the study reports). However, several studies were considered to be at high risk of ‘other bias’. Notably, the GEMINI 1 [36], GEMINI 2 [30], VISIBLE 1 [42], and PLANETCD [6] studies were considered to be at high risk of bias on the basis of only including patients in the maintenance phase if they had responded during the induction phase (i.e. at 6 weeks). This practice may potentially lead to overestimation of efficacy in the maintenance phase and overall safety, compared with studies in which both responders and non-responders were included in the maintenance phase. Therefore, the data relating to non-responders’ efficacy in infliximab (NOR-SWITCH and PLANETCD) and vedolizumab (GEMINI 1, GEMINI 2, and VISIBLE 1) during maintenance phase may not be generalized. Future studies should address the limitation of selectively progressing responders to the maintenance period, to permit transparent comparability of biological agents available for the treatment of IBD.

The level of heterogeneity observed within the meta-analyses was generally high, with *I*^*2*^ values exceeding 60% in a number of instances. This was likely influenced by the inclusion of studies with heterogeneous populations (e.g., TNFi-naïve patients and patients who had not responded adequately to prior TNFi therapy), as evidenced by the broad range of median disease durations reported across studies. It was not possible to conduct sensitivity analyses to address the source of heterogeneity due to small amount of available data. Likewise, the head-to-head trial is in need to address biases among the population and different study designs.

Several systematic reviews have examined the efficacy and/or safety of infliximab and vedolizumab in IBD. Our results confirm a prior comparative effectiveness and safety study in CD [45]. A study by Singh et al. (2018) concluded that infliximab was ranked highest among biological therapies for induction and maintenance of clinical remission [45]. When comparing infliximab and vedolizumab in TNF-naïve patients, infliximab yielded significantly better clinical response rates during the induction phase (Odds ratio (OR) 95% confidence interval [CI]: 0.08 [0.02 − 0.36]) and numerical advantages in clinical remission rates during both induction and maintenance phases (OR [95% CI]: induction 0.46 [0.16 − 1.26], maintenance 0.81 [0.39 − 1.67]). Consistent with these findings, the present study demonstrated the similar patterns of outcomes even when we included the most recent data from infliximab and vedolizumab studies (i.e., VISIBLE, VARSITY, the CT-P13 SC trial).

In UC, two network meta-analyses and one meta-analysis were conducted. The most recent network meta-analysis by Zhou et al. (2021) found no significant difference between vedolizumab and infliximab on clinical response [18]. Zhou and colleagues also found no difference between the treatments in clinical remission rates during induction phase [18], while Singh et al. reported a better clinical remission rate of infliximab in biologic-naïve patients (OR [95% CI]: clinical remission 0.62 [0.34 − 1.15]) [46]. The present study is in line with the clinical remission results of the past studies.

According to Zhou, endoscopic improvement rates were higher in infliximab compared to vedolizumab in biologic-naïve patients during induction therapy in UC (OR [95% CI]: 0.76 [0.42 − 1.37]) [46]; however, research by Cholapranee et al. (2017) reported that vedolizumab had higher mucosal healing rates than infliximab in the induction phase (OR [95% CI]: 0.63 [0.29 − 1.41]) [17]. Nevertheless, vedolizumab resulted in lower rates of mucosal healing compared to infliximab during the maintenance phase (OR [95% CI]: 1.17 [0.35–3.84]) [17]. The mucosal healing rates were numerically similar in the maintenance phase in the current study which disagrees to the prior meta-analyses. The reason for such a discrepancy may be due to the fact that the timeframe of our interest was different from the other studies.

Concerning the safety outcomes in patients with UC, Zhou and colleagues found that vedolizumab resulted in fewer occurrences of adverse events than infliximab (relative risk (RR): 0.79 [0.62 − 0.94]) [18]. Although insignificant, the serious adverse events were lower in vedolizumab; and a lower risk of infections was found in infliximab during maintenance therapy (RR [95% CI]: serious adverse event 1.12 [0.58 − 2.14], infection 0.80 [0.48 − 1.34]). The results conform to our safety outcomes, but in the current study, the proportions of patients experiencing serious adverse event and serious infection were comparable between the treatments with overlapping 95% CIs.

Narula and colleagues reported a post-hoc analysis of three UC clinical trial programmes, to compare the efficacy of infliximab and vedolizumab in patients with moderate-to-severe biologic-naïve UC. Broadly in agreement with the findings of the present review, the authors reported higher 1-year rates of clinical remission (corticosteroid free) and endoscopic remission with infliximab in comparison with vedolizumab, although the agents appeared to have similar efficacy in clinical symptom improvement [27].

Strengths of the present review process include prospective registration of the protocol (as documented on PROSPERO [20], comprehensive electronic searches and assessment of the included studies for risk of bias using gold-standard methods. Potential limitations of the review process include that a study was incorporated from outside of the prespecified time limits (to replace a recent article reporting data from the ACT 1 and ACT 2 studies); only articles published from 2010 onwards were used to ensure inclusion of studies relevant to current treatment practices, although we note that clinical practice continually evolves. For example, more recently, higher 10 mg/kg doses of infliximab are used in severe cases of IBD, and trough levels are actively monitored [47]. Such practices would tend to favour infliximab over vedolizumab, both in terms of observed efficacy and safety (e.g., monitoring of trough levels helps to reduce the risk of infusion reaction during induction phase and loss of response).

During the review process, we also noted that several studies were not registered, and results were thus untraceable, potentially leading to omission of some relevant data. Furthermore, some studies were excluded from the present review because the timepoints assessed did not match those prespecified in the review protocol. This omission of potentially valuable data highlights the need to standardise future study designs. The present review did not assess longer-term follow-up (i.e., beyond 1 year) despite a sustained response being important to patients. Longer-term follow-up in larger real-world cohorts may also be more relevant to analyse safety.

Finally, the included studies enrolled different proportions of patients with previous biological treatment failure (potentially accounting for between-study differences in efficacy and accounting for some of the observed heterogeneity). Different proportions of TNFi-experienced patients in vedolizumab treatment groups compared to infliximab treatment group, which consists of TNF-naïve patients only. For instance, taking the VARSITY trial into account exhibited different results for vedolizumab. The VARSITY trial is a head-to-head trial that compared the efficacy and safety between adalimumab and vedolizumab in TNF-naïve patients with UC. Higher clinical response and remission rates were achieved during induction on the VARSITY trial in comparison with GEMINI 1 (Fig. 2A, Additional file 1: Fig. 11). On the other hand, considerably lower rates of mucosal healing and clinical remission were found in the VARSITY trial compared with the VISIBLE and GEMINI 1 during the maintenance phase (Fig. 2B, Additional file 1: Fig. 14). Largely accounting for the fact that the VISIBLE and GEMINI 1 studies included Week 6 responders only to the maintenance phase, while the VARSITY trial included patients regardless of Week 6 responsiveness.

Despite limitations in the evidence, the present systematic review represents an up-to-date evaluation of data from RCTs of infliximab and vedolizumab in IBD, capturing important new data from recently published studies.

## Conclusions

Indirect comparison of infliximab and vedolizumab based on RCT data for the treatment of patients with IBD demonstrated that infliximab has significantly better efficacy in the induction phase, and comparable efficacy during the maintenance phase. A comparable safety profile including serious adverse event and serious infection between infliximab and vedolizumab was found over a year.

## Supplementary Information

**Additional file1: Table S1**. Characteristics of the included studies (detailed). **Table S2**. Baseline characteristics of participants with CD. **Table S3**. Baseline characteristics of participants with UC. **Figure S1**. Forest plots showing the proportion of patients with Crohn’s disease achieving a CDAI-70 response during the induction phase with infliximab (upper plot) or vedolizumab (lower plot). **Figure S2**. Forest plots showing the proportion of with Crohn’s disease achieving a CDAI-100 response during the induction phase with infliximab (upper plot) or vedolizumab (lower plot). **Figure S3**. Forest plots showing the proportion of patients with Crohn’s disease achieving a CDAI-70 response during the maintenance phase with infliximab. **Figure S4**. Forest plots showing the proportion of patients with Crohn’s disease achieving a CDAI-100 response during the maintenance phase with infliximab (upper plot) or vedolizumab (lower plot). **Figure S5**. Forest plots showing the proportion of patients with Crohn’s disease experiencing any adverse event with infliximab (upper plot) or vedolizumab (lower plot). **Figure S6**. Forest plots showing the proportion of patients with Crohn’s disease experiencing any serious adverse event with infliximab (upper plot) or vedolizumab (lower plot). **Figure S7**. Forest plots showing the proportion of patients with Crohn’s disease experiencing any infection with infliximab (upper plot) or vedolizumab (lower plot). **Figure S8**. Forest plots showing the proportion of patients with Crohn’s disease experiencing any serious infection with infliximab (upper plot) or vedolizumab (lower plot). **Figure S9**. Forest plots showing the proportion of patients with Crohn’s disease who discontinued due to adverse events in the infliximab (upper plot) or vedolizumab (lower plot) treatment arms. **Figure S10**. Forest plots showing the proportion of patients with Crohn’s disease who discontinued due to lack of efficacy in the infliximab treatment arm. **Figure S11**. Forest plots showing the proportion of patients with ulcerative colitis achieving a clinical response during the induction phase with infliximab (upper plot) or vedolizumab (lower plot). **Figure S12**. Forest plot showing the proportion of patients with ulcerative colitis achieving mucosal healing during the induction phase with infliximab. **Figure S13**. Forest plots showing the proportion of patients with ulcerative colitis achieving a clinical response during the maintenance phase with infliximab (upper plot) or vedolizumab (lower plot). **Figure S14**. Forest plots showing the proportion of patients with ulcerative colitis achieving mucosal healing during the maintenance phase with infliximab (upper plot) or vedolizumab (lower plot). **Figure S15**. Forest plots showing the proportion of patients with ulcerative colitis experiencing any adverse event with infliximab (upper plot) or vedolizumab (lower plot). **Figure S16**. Forest plots showing the proportion of patients with ulcerative colitis experiencing any serious adverse event with infliximab (upper plot) or vedolizumab (lower plot). **Figure S17**. Forest plots showing the proportion of patients with ulcerative colitis experiencing any infection with infliximab (upper plot) or vedolizumab (lower plot). **Figure S18**. Forest plots showing the proportion of patients with ulcerative colitis experiencing any serious infection with infliximab (upper plot) or vedolizumab (lower plot). **Figure S19**. Forest plots showing the proportion of patients with ulcerative colitis who discontinued due to adverse events with infliximab (upper plot) or vedolizumab (lower plot). **Figure S20**. Forest plots showing the proportion of patients with ulcerative colitis who discontinued due to lack of efficacy with vedolizumab.

## Data Availability

All data generated or analysed during this study are included in this published article and its supplementary information files.

## Abbreviations

AE: Adverse event
CD: Crohn’s disease
CDAI: Crohn’s disease activity index
CI: Confidence interval
IBD: Inflammatory bowel disease
IV: Intravenous
PRISMA: Preferred reporting items for systematic reviews and meta-analyses
Q2W: Every 2 weeks
Q8W: Every 8 weeks
RCT: Randomised controlled trial
SAE: Serious adverse event
SC: Subcutaneous
SmPC: Summary of product characteristics
TNFi: Tumour necrosis factor-*α* inhibitor
UC: Ulcerative colitis
