# Supplementary material for: Comparative efficacy and safety of infliximab and vedolizumab therapy in patients with inflammatory bowel disease: a systematic review and meta-analysis
Source: BMC Gastroenterol. 2022 Jun 8;22:291. doi: 10.1186/s12876-022-02347-1 (PMC9178865; doi:10.1186/s12876-022-02347-1)
Supplement: Supplementary file 1 — Additional file1: Table S1. Characteristics of the included studies (detailed). Table S2. Baseline characteristics of participants with CD. Table S3. Baseline characteristics of participants with UC. Figure S1. Forest plots showing the proportion of patients with Crohn’s disease achieving a CDAI-70 response during the induction phase with infliximab (upper plot) or vedolizumab (lower plot). Figure S2. Forest plots showing the proportion of with Crohn’s disease achieving a CDAI-100 response during the induction phase with infliximab (upper plot) or vedolizumab (lower plot). Figure S3. Forest plots showing the proportion of patients with Crohn’s disease achieving a CDAI-70 response during the maintenance phase with infliximab. Figure S4. Forest plots showing the proportion of patients with Crohn’s disease achieving a CDAI-100 response during the maintenance phase with infliximab (upper plot) or vedolizumab (lower plot). Figure S5. Forest plots showing the proportion of patients with Crohn’s disease experiencing any adverse event with infliximab (upper plot) or vedolizumab (lower plot). Figure S6. Forest plots showing the proportion of patients with Crohn’s disease experiencing any serious adverse event with infliximab (upper plot) or vedolizumab (lower plot). Figure S7. Forest plots showing the proportion of patients with Crohn’s disease experiencing any infection with infliximab (upper plot) or vedolizumab (lower plot). Figure S8. Forest plots showing the proportion of patients with Crohn’s disease experiencing any serious infection with infliximab (upper plot) or vedolizumab (lower plot). Figure S9. Forest plots showing the proportion of patients with Crohn’s disease who discontinued due to adverse events in the infliximab (upper plot) or vedolizumab (lower plot) treatment arms. Figure S10. Forest plots showing the proportion of patients with Crohn’s disease who discontinued due to lack of efficacy in the infliximab treatment arm. Figure S11. Forest plots showing th [file 12876_2022_2347_MOESM1_ESM.docx]

# Supplementary materials

**Title: Comparative Efficacy and Safety of Infliximab and Vedolizumab Therapy in Patients with Inflammatory Bowel Disease: A Systematic Review and Meta-analysis**

**Authors:** Laurent Peyrin Biroulet, Perttu Arkkila, Alessandro Armuzzi, Silvio Danese, Jordi Guardiola Capon, Jørgen Jahnsen, Charles Lees, Edouard Louis, Milan Lukáš, Walter Reinisch, Xavier Roblin, Minyoung Jang, Han Geul Byun, Dong-Hyeon Kim, Sung Jeong Lee, Raja Atreya

**Corresponding author:** Raja Atreya, Medical Department 1, University Hospital Erlangen, Friedrich-Alexander-University of Erlangen-Nürnberg, Ulmenweg 18, 91054 Erlangen, Germany. Email: [raja.atreya@uk-erlangen.de](mailto:raja.atreya@uk-erlangen.de\\)

Table of Contents

[Supplementary materials 1](#_Toc101343254)

[Table of Contents 2](#_Toc101343255)

[Supplementary methods 4](#_Toc101343256)

[Sample PubMed search strategy for Crohn’s disease cohort 4](#_Toc101343257)

[Sample PubMed search strategy for ulcerative colitis cohort 5](#_Toc101343258)

[Supplementary results 6](#_Toc101343259)

[Supplementary Table 1 Characteristics of the included studies (detailed) 6](#_Toc101343260)

[Supplementary Table 2. Baseline characteristics of participants with CD 16](#_Toc101343261)

[Supplementary Table 3. Baseline characteristics of participants with UC 17](#_Toc101343262)

[Supplementary Figure 1. Forest plots showing the proportion of patients with Crohn’s disease achieving a CDAI-70 response during the induction phase with infliximab (upper plot) or vedolizumab (lower plot) 18](#_Toc101343263)

[Supplementary Figure 2. Forest plots showing the proportion of with Crohn’s disease achieving a CDAI-100 response during the induction phase with infliximab (upper plot) or vedolizumab (lower plot) 19](#_Toc101343264)

[Supplementary Figure 3. Forest plots showing the proportion of patients with Crohn’s disease achieving a CDAI-70 response during the maintenance phase with infliximab 20](#_Toc101343265)

[Supplementary Figure 4. Forest plots showing the proportion of patients with Crohn’s disease achieving a CDAI-100 response during the maintenance phase with infliximab (upper plot) or vedolizumab (lower plot) 21](#_Toc101343266)

[Supplementary Figure 5. Forest plots showing the proportion of patients with Crohn’s disease experiencing any adverse event with infliximab (upper plot) or vedolizumab (lower plot) 22](#_Toc101343267)

[Supplementary Figure 6. Forest plots showing the proportion of patients with Crohn’s disease experiencing any serious adverse event with infliximab (upper plot) or vedolizumab (lower plot) 23](#_Toc101343268)

[Supplementary Figure 7. Forest plots showing the proportion of patients with Crohn’s disease experiencing any infection with infliximab (upper plot) or vedolizumab (lower plot) 24](#_Toc101343269)

[Supplementary Figure 8. Forest plots showing the proportion of patients with Crohn’s disease experiencing any serious infection with infliximab (upper plot) or vedolizumab (lower plot) 25](#_Toc101343270)

[Supplementary Figure 9. Forest plots showing the proportion of patients with Crohn’s disease who discontinued due to adverse events in the infliximab (upper plot) or vedolizumab (lower plot) treatment arms 26](#_Toc101343271)

[Supplementary Figure 10. Forest plots showing the proportion of patients with Crohn’s disease who discontinued due to lack of efficacy in the infliximab treatment arm 27](#_Toc101343272)

[Supplementary Figure 11. Forest plots showing the proportion of patients with ulcerative colitis achieving a clinical response during the induction phase with infliximab (upper plot) or vedolizumab (lower plot) 28](#_Toc101343273)

[Supplementary Figure 12. Forest plot showing the proportion of patients with ulcerative colitis achieving mucosal healing during the induction phase with infliximab 29](#_Toc101343274)

[Supplementary Figure 13. Forest plots showing the proportion of patients with ulcerative colitis achieving a clinical response during the maintenance phase with infliximab (upper plot) or vedolizumab (lower plot) 30](#_Toc101343275)

[Supplementary Figure 14. Forest plots showing the proportion of patients with ulcerative colitis achieving mucosal healing during the maintenance phase with infliximab (upper plot) or vedolizumab (lower plot) 31](#_Toc101343276)

[Supplementary Figure 15. Forest plots showing the proportion of patients with ulcerative colitis experiencing any adverse event with infliximab (upper plot) or vedolizumab (lower plot) 32](#_Toc101343277)

[Supplementary Figure 16. Forest plots showing the proportion of patients with ulcerative colitis experiencing any serious adverse event with infliximab (upper plot) or vedolizumab (lower plot) 33](#_Toc101343278)

[Supplementary Figure 17. Forest plots showing the proportion of patients with ulcerative colitis experiencing any infection with infliximab (upper plot) or vedolizumab (lower plot) 34](#_Toc101343279)

[Supplementary Figure 18. Forest plots showing the proportion of patients with ulcerative colitis experiencing any serious infection with infliximab (upper plot) or vedolizumab (lower plot) 35](#_Toc101343280)

[Supplementary Figure 19. Forest plots showing the proportion of patients with ulcerative colitis who discontinued due to adverse events with infliximab (upper plot) or vedolizumab (lower plot) 36](#_Toc101343281)

[Supplementary Figure 20. Forest plots showing the proportion of patients with ulcerative colitis who discontinued due to lack of efficacy with vedolizumab 37](#_Toc101343282)

[References 38](#_Toc101343283)

# Supplementary methods

## Sample PubMed search strategy for Crohn’s disease cohort

(‘randomized controlled trial*’[pt] OR ‘controlled clinical trial’[pt] OR ‘randomized’[tiab] OR ‘placebo’[tiab] OR ‘drug therapy’[sh] OR ‘randomly’[tiab] OR ‘trial’[tiab] OR ‘group*’[tiab]) AND ((‘crohn disease activity’ OR ‘cdai’ OR ‘crohn$ disease activity index’ OR ‘crohn’s disease activity index’ OR ‘crohns disease activity index’ OR ‘cdai#70’ OR ‘cdai70’ OR ‘cdai#100’ OR ‘cdai100’ OR ‘remission’ OR ‘disease regression’ OR ‘disease remission’ OR ‘regression, disease’ OR ‘remission’ OR ‘remission induction’ OR ‘remission rate’ OR ‘remission, spontaneous’ OR ‘spontaneous regression’ OR ‘endoscopic response’ OR ‘simplified endoscopic activity score for crohn*’ OR ‘ses cd’ OR ‘short inflammatory bowel disease questionnaire’ OR ‘sibdq’ OR ‘response’ OR ‘response rate’ OR ‘mucosal healing’ OR ‘CR#70’ OR ‘CR70’ OR ‘CR#100’ OR ‘CR100’) OR (‘safety’ OR ‘adverse event’ OR ‘adverse effect’ OR ‘adverse reaction’ OR ‘infection’ OR ‘immunogenicity’ OR ‘immune response’ OR ‘tolerability’ OR discontinu* OR complian* OR adheren* OR ‘lack of efficacy’ OR ‘loss of response’ OR ‘disease worsening’ OR ‘worsening disease’ OR ‘disease worse*’)) AND (Crohn* OR CD) AND (‘infliximab’ OR ‘remicade’ OR ‘remsima’ OR ‘CT-P13’ OR ‘inflectra’ OR ‘infliximab#dyyb’ OR ‘CTP13’ OR ‘CT#P13’ OR ‘Infliximab#abda’ OR ‘Renflexis’ OR ‘Flixabi’ OR ‘SB2’ OR ‘SB#2’ OR ‘infliximab#qbtx’ OR ‘Ixifi’ OR ‘Zessly’ OR ‘PF#06438179’ OR ‘PF06438179’ OR ‘PF#6438179’ OR ‘PF6438179’ OR ‘GP1111’ OR ‘GP#1111’ OR ‘infliximab#axxq’ OR ‘Avsola’ OR ‘ABP710’ OR ‘ABP#710’ OR ‘vedolizumab’ OR ‘entyvio’ OR ‘MLN0002’ OR ‘MLN#0002’) AND (‘week’ OR ‘week*’)

## Sample PubMed search strategy for ulcerative colitis cohort

(‘infliximab’ OR ‘remicade’ OR ‘remsima’ OR ‘CT-P13’ OR ‘inflectra’ OR ‘infliximab#dyyb’ OR ‘CTP13’ OR ‘CT#P13’ OR ‘Infliximab#abda’ OR ‘Renflexis’ OR ‘Flixabi’ OR ‘SB2’ OR ‘SB#2’ OR ‘infliximab#qbtx’ OR ‘Ixifi’ OR ‘Zessly’ OR ‘PF#06438179’ OR ‘PF06438179’ OR ‘PF#6438179’ OR ‘PF6438179’ OR ‘GP1111’ OR ‘GP#1111’ OR ‘infliximab#axxq’ OR ‘Avsola’ OR ‘ABP710’ OR ‘ABP#710’ OR ‘vedolizumab’ OR ‘entyvio’ OR ‘MLN0002’ OR ‘MLN#0002’) AND (‘ulcerative colitis’ OR UC) AND (‘mayo score’ OR ‘mayo’ OR ‘mayo scoring system’ OR ‘mss’ OR ‘blood faeces’ OR ‘blood feces’ OR ‘blood stool’ OR ‘blood stools’ OR ‘bloody stool’ OR ‘bloody stools’ OR ‘faeces, blood’ OR ‘feces, blood’ OR ‘haematochezia’ OR ‘haemorrhage, rectum’ OR ‘hematochezia’ OR ‘hemorrhage, rectum’ OR ‘proctorrhagia’ OR ‘rectal bleeding’ OR ‘rectal blood loss’ OR ‘rectal haemorrhage’ OR ‘rectal hemorrhage’ OR ‘rectocolic bleeding’ OR ‘rectorrhagia’ OR ‘rectum bleeding’ OR ‘rectum blood loss’ OR ‘rectum haemorrhage’ OR ‘rectum hemorrhage’ OR ‘stool, blood’ OR ‘mucosal healing’ OR ‘short inflammatory bowel disease questionnaire’ OR ‘sibdq’ OR ‘remission’ OR ‘disease regression’ OR ‘disease remission’ OR ‘regression, disease’ OR ‘remission induction’ OR ‘remission rate’ OR ‘remission, spontaneous’ OR ‘spontaneous regression’ OR ‘response’ OR ‘response rate’ OR ‘safety’ OR ‘adverse event’ OR ‘adverse effect’ OR ‘adverse reaction’ OR ‘infection’ OR ‘immunogenicity’ OR ‘immune response’ OR ‘tolerability’ OR discontinu* OR complian* OR adheren* OR ‘lack of efficacy’ OR ‘loss of response’ OR ‘disease worsening’ OR ‘worsening disease*’ OR ‘disease worse’) AND (‘randomized controlled trial’[pt] OR ‘controlled clinical trial’[pt] OR ‘randomized’[tiab] OR ‘placebo’[tiab] OR ‘drug therapy’[sh] OR ‘randomly’[tiab] OR ‘trial’[tiab] OR ‘groups’[tiab]) AND (‘week’ OR ‘week*’)

# Supplementary results

## Supplementary Table 1 Characteristics of the included studies (detailed)

| **RCT (trial name)^Reference^** | **Country (no. of centres)** | **Indication** | **Patients** | **Randomised arms** | ***N*** | **Arms contributing  data to meta-analyses** | **Study design** |
| --- | --- | --- | --- | --- | --- | --- | --- |
| **Infliximab studies** | | | | | | | |
| NCT00094458 (SONIC)[1] | Global (92 centres)  CD | | Age ≥21 years  Disease duration ≥6 weeks  CDAI 220–450 points and either corticosteroid dependent, being considered for second course of corticosteroids within 12 months, or no response to mesalamine or budesonide  Exclusion: any prior treatment with AZA, 6-MP, MTX or TNFi | Reference IFX 5 mg/kg IV infusion (Weeks 0, 2, 6, then Q8W) plus oral placebo QD | 169 | IFX 5 mg/kg IV;  IFX 5 mg/kg + AZA 2.5 mg/kg QD | 30-week double-blind period + optional double-blind 20-week extension  Randomisation 1:1:1 (stratification factors: centre, duration of CD [<3 or ≥3 years], systemic corticosteroid dose [<20 or ≥20 mg of prednisone equivalent  daily])  Oral mesalamine could be continued at a stable dose; systemic corticosteroids permitted until Week 14, then tapered; budesonide could be continued or decreased until Week 14 (maximum 9 mg daily), then tapered |
|  |  |  |  | Oral AZA 2.5 mg/kg QD plus placebo IV infusion (Weeks 0, 2, 6, then Q8W) | 170 |  |  |
|  |  |  |  | Reference IFX 5 mg/kg IV (Weeks 0, 2, 6, then Q8W) plus oral AZA 2.5 mg/kg QD | 169 |  |  |
| NCT02096861 (PLANET CD)[2] | Global (58 centres) | CD | Age 18–75 years  Disease duration ≥12 weeks  CDAI 220–450 and had not responded to, were intolerant of, or had contraindications for non-biological treatments for CD  Exclusion: any prior treatment with TNFi, prior biologics for CD | CT-P13 5 mg/kg IV (Weeks 0, 2, 6, then Q8W) | 56 | CT-P13 5 mg/kg IV; CT-P13→IFX; IFX 5 mg/kg IV; IFX→CT-P13 | 54-week double-blind study; EOS visit after 8 weeks  Week 14 non-responders discontinued study treatment  Randomisation 1:1:1:1 (stratification factors: region [European or non-  European], history of treatment with immunomodulators,  disease duration [<3 or ≥3 years])  Stable doses of 5-ASA, antibiotics, AZA, 6-MP or MTX could be continued; corticosteroids (≤30 mg/day prednisone equivalent) could be tapered after Week 6 |
|  |  |  |  | CT-P13 5 mg/kg IV (Weeks 0, 2, 6, then Q8W to Week 22), then reference IFX 5 mg/kg Q8W | 55 |  |  |
|  |  |  |  | Reference IFX 5 mg/kg IV (Weeks 0, 2, 6, then Q8W) | 54 |  |  |
|  |  |  |  | Reference IFX 5 mg/kg IV (Weeks 0, 2, 6, then Q8W to Week 22), then CT-P13 5 mg/kg Q8W | 55 |  |  |
| NCT02148640 (NOR-SWITCH)[3,4] | Norway (40 centres) | CD, UC | Age >18 years  Diagnosis of CD or UC  Stable treatment with IFX for ≥6 months | Continue reference IFX (main study); regimen continued from pre-randomisation | CD: 78  UC: 47 | Continue IFX; Switch to  CT-P13 | 52-week double-blind main study + 26-week open extension  Randomisation 1:1 (main study; stratification factors: diagnosis)  Initiation of systemic corticosteroids, immunosuppressant or any drug affecting disease stability was not permitted within 2 months prior to randomization |
|  |  |  |  | Switch to CT-P13 (main study); regimen continued from pre-randomisation | CD: 77  UC: 46 |  |  |
|  |  |  |  | Continue reference IFX (main study), then switch to CT-P13 (extension); regimen continued from pre-randomisation | 100 (62 CD, 38 UC) |  |  |
|  |  |  |  | Switch to CT-P13 (main study) and continue in extension; regimen continued from pre-randomisation | 107 (65 CD, 42 UC) |  |  |
| NCT02883452 (CT-P13 SC trial)[5] | Global (50 centres) | CD, UC | Age 18–75 years  Disease duration ≥3 months  Active CD (CDAI 220–450) or active UC (total Mayo score 6–12 points with endoscopic sub-score ≥2)  Patients had not responded to an adequate course of conventional therapy  Exclusion: any prior TNFi, prior biologics for CD/UC, tuberculosis exposure/diagnosis | CT-P13 5 mg/kg IV (Weeks 0, 2, 6, then Q8W until Week 22), followed by CT-P13 120 mg (patients <80 kg) or CT-P13 240 mg (patients ≥80 kg) SC Q2W | 65 (CD 25, UC 40) | IV Q8W→SC Q2W; IV Weeks 0, 2→SC Q2W | 54-week open-label study (follow-up to Week 56)  Randomisation 1:1 (stratification factors: current treatment with AZA or 6-MP or MTX [used or not used]; clinical response at Week 6 [responder or non-responder]; body weight at Week 6 [<80 or ≥80 kg]); disease [UC or CD])  Stable doses of thiopurines or MTX could be continued throughout |
|  |  |  |  | CT-P13 5 mg/kg IV (Weeks 0, 2), followed by CT-P13 120 mg (patients <80 kg) or CT-P13 240 mg (patients ≥80 kg) SC Q2W from Week 6 | 66 (CD 28, UC 38) |  |  |
| NCT00036439 (ACT1)[6] | Global (62 centres) | UC | Adult patients with established UC diagnosis  Mayo score 6–12 points and endoscopic sub-score ≥2 despite concurrent corticosteroids with/without AZA or mercaptopurine  Exclusion: prior TNFi, rectally administered corticosteroids or 5-ASA within 2 weeks, positive tuberculin skin test | Reference IFX 5 mg/kg IV (Weeks 0, 2, 6, then Q8W) | 121 | IFX 5 mg/kg IV; IFX 10 mg/kg IV | 46-week double-blind phase (follow-up to Week 54)  Randomisation 1:1:1 (stratification factors: site, UC refractory to corticosteroid therapy [yes or no])  Stable doses of concomitant medications continued; corticosteroids tapered after Week 8 |
|  |  |  |  | Reference IFX 10 mg/kg IV (Weeks 0, 2, 6, then Q8W) | 122 |  |  |
|  |  |  |  | Placebo IV (Weeks 0, 2, 6, then Q8W) | 121 |  |  |
| NCT00096655 (ACT2)[6] | Global (55 centres) | UC | Adult patients with established UC diagnosis  Mayo score 6–12 points and endoscopic sub-score ≥2 despite concurrent corticosteroids with/without AZA or mercaptopurine and 5-ASA  Exclusion: prior TNFi, rectally administered corticosteroids or 5-ASA within 2 weeks, positive tuberculin skin test | Reference IFX 5 mg/kg IV (Weeks 0, 2, 6, then Q8W) | 121 | IFX 5 mg/kg; IFX 10 mg/kg | 22-week double-blind phase (follow-up to Week 30)  Randomisation 1:1:1 (stratification factors: site, UC refractory to corticosteroid therapy [yes or no])  Stable doses of concomitant medications continued; corticosteroids tapered after Week 8 |
|  |  |  |  | Reference IFX 10 mg/kg IV (Weeks 0, 2, 6, then Q8W) | 120 |  |  |
|  |  |  |  | Placebo IV (Weeks 0, 2, 6, then Q8W) | 123 |  |  |
| **Vedolizumab studies** | | | | | | | |
| NCT00783692 (GEMINI 2)[7] | Global (285 centres) | CD | Age 18–80 years  Disease duration ≥3 months  CDAI 220–450 points and one of: CRP >2.87 mg/L; ≥3 large ulcers or ≥10 aphthous ulcers on colonoscopy; or FC >250 µg/g of stool with evidence of ulcers on CT, magnetic resonance enterography, small-bowel radiography or capsule endoscopy  No response or unacceptable side effects with ≥1 of glucocorticoids, immunosuppressive agents, or TNFi  Exclusions: prior vedolizumab, natalizumab, efalizumab or rituximab; adalimumab within 30 days; infliximab or certolizumab pegol within 60 days; patients with active or latent tuberculosis, cancer, or a stoma, >3 small-bowel resections, short-bowel syndrome, extensive colonic resection, intestinal stricture, or abdominal abscess | VDZ 300 mg IV (Weeks 0, 2, 6, then Q8W) | 154 | VDZ 300 mg IV Q8W (VDZ prior to TNFi, VDZ after TNFi failure) | 52-week study  Randomisation 3:2 to vedolizumab or placebo in double-blind induction trial (stratification factors: concomitant use of  glucocorticoids; concomitant use of immunosuppressive agents, prior use of TNFis, or both); additional patients enrolled for open-label vedolizumab induction therapy  Week 6 responders to vedolizumab randomised 1:1:1 for maintenance trial (non-responders received vedolizumab Q4W from Week 6)  Stable doses of oral prednisone or budesonide, immunosuppressive agents, mesalamine and antibiotics were permitted |
|  |  |  |  | VDZ 300 mg IV (Weeks 0, 2, 6, then Q4W) | 412 Week 6 non-responders; 154 Week 6 responders |  |  |
|  |  |  |  | VDZ 300 mg IV (Weeks 0, 2), followed by placebo IV | 153 |  |  |
|  |  |  |  | Placebo IV (Weeks 0, 2, 6, then Q8W) | 148 |  |  |
| NCT01224171 (GEMINI 3)[8] | Global (107 centres) | CD | Age 18–80 years  Disease duration ≥3 months (or ≥6 months if histopathology report unavailable)  CDAI 220–400 points and one of: CRP >2.87 mg/L, ulcerations on colonoscopy within 4 months, FC ≥250 µg/g of stool with features of active CD on small-bowel imaging  Inadequate response, loss of response, or intolerance to TNFi; immunosuppressives or corticosteroids within 5 years  Exclusion: prior vedolizumab, natalizumab, efalizumab, or rituximab; most prior malignancies; pregnancy/lactation or unstable/uncontrolled medical conditions | VDZ 300 mg IV (Weeks 0, 2, 6) | 209 | VDZ 300 mg IV (TNFi failure; TNFi naïve) | 10-week double-blind treatment period, followed by optional enrolment in long-term, open-label safety extension study  Randomisation 1:1 (stratification factors: previous TNFi status [failure/no experience], concomitant oral corticosteroid use [yes/no], concomitant immunosuppressive  use [yes/no])  Concomitant oral corticosteroids and immunosuppressives were permitted |
|  |  |  |  | Placebo IV (Weeks 0, 2, 6) | 207 |  |  |
| NCT00783718 (GEMINI 1)[9] | Global (211 centres) | UC | Age 18–80 years  Mayo score 6–12 with sigmoidoscopy sub-score ≥2; disease extending ≥15 cm from anal verge  Lack of response or unacceptable adverse events with ≥1 of glucocorticoids, AZA, 6-MP or TNFi  Exclusion: prior vedolizumab, natalizumab, efalizumab, or rituximab; TNFi within 60 days; toxic megacolon, abdominal abscess, stoma, symptomatic colonic stricture, prior colectomy, increased risk of infectious complications; clinically meaningful laboratory abnormalities; anticipated requirement for major surgery; colonic dysplasia or adenomas; malignant neoplasms; pregnancy/lactation or unstable/uncontrolled medical disorder | VDZ 300 mg IV (Days 1, 15, then Q8W) | 122 | VDZ 300 mg IV Q8W VDZ 300 mg IV Q4W | 52-week double-blind study  Randomisation 3:2 to vedolizumab or placebo in double-blind induction trial (stratification factors: concomitant glucocorticoid use [yes/no], concomitant immunosuppressive use [yes/no] or prior use of TNFi [yes/no experience]); additional patients enrolled for open-label vedolizumab induction therapy  Week 6 responders to vedolizumab randomised 1:1:1 for maintenance trial (stratification factors: cohort [1 vs 2], concomitant glucocorticoid use [yes/no], concomitant immunosuppressive or prior TNFi use [yes/no]); non-responders received vedolizumab Q4W from Week 6  Stable doses of mesalamine, prednisone (≤30 mg/day or equivalent) or immunosuppressives could be continued |
|  |  |  |  | VDZ 300 mg IV (Days 1, 15, then Q4W) | 125 Week 6 responders |  |  |
|  |  |  |  | VDZ 300 mg IV (Days 1, 15), followed by placebo | 126 |  |  |
|  |  |  |  | Placebo IV (Days 1, 15) | 149 |  |  |
| NCT02497469 (VARSITY)[10] | Global (245 centres) | UC | Age 18–85 years  Disease duration ≥3 months  Total Mayo score 6–12 with endoscopic sub-score ≥2 and ≥15 cm colonic involvement  No response or loss of response to conventional treatment  Exclusion: prior TNFi (unless discontinued for a documented reason other than safety [except for adalimumab]), prior vedolizumab | VDZ 300 mg IV (Day 1, Weeks 2, 6, then Q8W) | 383 | VDZ 300 mg IV Q8W | 52-week double-blind study (final safety follow-up at Week 68) Randomisation 1:1 to adalimumab or vedolizumab (stratification factors: concomitant oral corticosteroid use [yes/no], prior TNFi use [yes/no])  Stable doses of an aminosalicylate or immunomodulator continued, stable corticosteroids were continued until Week 6, then tapered |
|  |  |  |  | Adalimumab 160 mg SC induction (Days 1 and/or 2), followed by 80 mg SC (Week 2), then 40 mg Q2W | 386 |  |  |
| NCT02611830 (VISIBLE 1)[11] | Global (141 centres) | UC | Age 18–80 years  Moderately to severely active UC for ≥6 months  Total Mayo score 6–12 with endoscopic sub-score ≥2 and ≥15 cm of involved colon  Inadequate response to, loss of response to, or intolerance to ≥1 corticosteroid, immunomodulator or TNFi  Exclusion: prior anti-integrin, anti-MAdCAM-1, or rituximab therapy, biologics within 60 days or 5 half-lives, non-biological therapies within 30 days or 5 half-lives; abdominal abscess; toxic megacolon; subtotal or total colectomy; unresected adenomatous colonic polyps; colonic mucosal dysplasia | VDZ 300 mg IV (Weeks 0, 2), followed by VDZ 108 mg SC Q2W | 106 | VDZ IV → VDZ SC; VDZ 300 mg IV Q8W | Open-label induction therapy, followed by response assessment  Week 6 responders randomised 2:1:1 (stratification factors: stratification by concomitant corticosteroid use, clinical remission status at Week 6; previous anti-TNF failure or concomitant immunomodulator use)  Week 6 non-responders received vedolizumab 300 mg IV and assessed for response at Week 14; responders could enter open-label extension study to Week 52 (final safety follow-up at Week 68); non-responders discontinued  Stable oral mesalamine, AZA, 6-MP continued; stable corticosteroids continued until Week 6, then tapered |
|  |  |  |  | VDZ 300 mg IV (Weeks 0, 2), then Q8W | 54 |  |  |
|  |  |  |  | VDZ 300 mg IV (Weeks 0, 2), then placebo | 56 |  |  |

Abbreviation: 5-ASA, 5-aminosalicylates; 6-MP, 6-mercaptopurine; AZA, azathioprine; CD, Crohn’s disease; CDAI, Crohn’s Disease Activity Index; CRP, C-reactive protein; CT, computed tomography; EOS, end of study; FC, faecal calprotectin; IFX, infliximab; IV, intravenous; MAdCAM-1, mucosal addressin cell adhesion molecule 1; MTX, methotrexate; Q2W, every 2 weeks; Q4W, every 4 weeks; Q8W, every 8 weeks; QD, once daily; RCT, randomised controlled trial; SC, subcutaneous; TNFi, tumour necrosis factor inhibitor; UC, ulcerative colitis; VDZ, vedolizumab

## Supplementary Table 2. Baseline characteristics of participants with CD

| **RCT (trial name)^Reference^** | **Randomised arms included in meta-analyses** | ***N*** | **Age, median (range), years** | **Sex, % female** | **Body weight,**  **median (range), kg** | **Disease duration,**  **mean (SD), years** |
| --- | --- | --- | --- | --- | --- | --- |
| NCT00094458  (SONIC)[1] | Infliximab  Infliximab + azathioprine | 169  169 | 35.0 (18–80)  34.0 (19–68) | 50  48 | 68.9 (NA)  72.0 (NA) | 2.2 (NA)^a^  2.2 (NA)^a^ |
| NCT02096861  (PLANET CD)[2] | CT-P13  Infliximab | 111  109 | 35.0 (26–46)  32.0 (24–45) | 43  45 | NR  NR | 4.2 (4.6)  5.3 (7.3) |
| NCT02148640  (NOR-SWITCH)[3] | CT-P13  Infliximab | 77  78 | 39.5 (14.2)^b^  38.0 (13.4)^b^ | 39  42 | NR  NR | 14.3 (8.5)  12.8 (9.0) |
| NCT02883452^c^ (CT-P13 SC trial)[5] | CT-P13 SC  CT-P13 IV | 66  65 | 33.0 (18–69)  36.0 (18–70) | 46  46 | 66.1 (45.2–117.0)  69.0 (43.2–116.2) | 5.7 (6.0)  5.9 (6.3) |
| NCT00783692 (GEMINI 2)[7] | Vedolizumab | 967 | 35.7 (11.9)^b^ | 53 | 69.9 (19.5)^b^ | 9.2 (7.8) |
| NCT01224171 (GEMINI 3)[8] | Vedolizumab | 209 | 36.9 (20–69) | 56 | 69.5 (40–144)^d^ | 8.4 (0.3–41.8)^a^ |

^a^Data are median (range)
^b^Data are mean (SD)
^c^Data shown are for CD + UC (CT-P13 SC: 42% CD, 58% UC; CT-P13 IV: 38% CD, 62% UC)
^d^Data are mean (range)

Abbreviation: CD, Crohn’s disease; IV, intravenous; NA, not available; NR, not reported; RCT, randomised controlled trial; SC, subcutaneous; SD, standard deviation; UC, ulcerative colitis

## Supplementary Table 3. Baseline characteristics of participants with UC

| **RCT (trial name)^Reference^** | **Randomised arms included in meta-analyses** | ***N*** | **Age, median (range), years** | **Sex, % female** | **Body weight, median (range), kg** | **Disease duration,**  **mean (SD), years** |
| --- | --- | --- | --- | --- | --- | --- |
| NCT02148640  (NOR-SWITCH)[3] | CT-P13  Infliximab | 46  47 | 44.4 (14.8)^a^  45.8 (14.14)^a^ | 30  38 | NR  NR | 11.5 (7.5)  11.2 (9.2) |
| NCT02883452^b^ (CT-P13 SC trial)[5] | CT-P13 SC  CT-P13 IV | 66  65 | 33.0 (18–69)  36.0 (18–70) | 46  46 | 66.1 (45.2–117.0)  69.0 (43.2–116.2) | 5.7 (6.0)  5.9 (6.3) |
| NCT00036439  (ACT1)[6] | Infliximab (5 mg)  Infliximab (10 mg) | 121  122 | 42.4 (14.3)^a^  41.8 (14.9)^a^ | 36  41 | 80.0 (17.8)^a^  76.9 (17.1)^a^ | 5.9 (5.4)  8.4 (8.1) |
| NCT00096655  (ACT2)[6] | Infliximab (5 mg)  Infliximab (10 mg) | 121  120 | 40.5 (13.1)^a^  40.3 (13.3)^a^ | 37  43 | 78.4 (17.8)^a^  79.6 (20.6)^a^ | 6.7 (5.3)  6.5 (5.8) |
| NCT00783718  (GEMINI 1)[9] | Vedolizumab | 746 | 40.1 (13.2)^a^ | 42 | 73.6 (18.7)^a^ | 6.8 (6.2) |
| NCT02497469  (VARSITY)[10] | Vedolizumab | 385 | 40.8 (13.7)^a^ | 39 | 72.7 (17.0)^a^ | 7.3 (7.2) |
| NCT02611830  (VISIBLE 1)[11] | Vedolizumab SC  Vedolizumab IV | 106  54 | 38.1 (13.1)  41.6 (14.1) | 39  43 | 71.6 (17.2)  77.0 (16.9) | 8.0 (6.2)  8.2 (5.9) |

^a^Data are mean (SD)
^b^Data shown are for CD + UC (CT-P13 SC: 42% CD, 58% UC; CT-P13 IV: 38% CD, 62% UC)

Abbreviation: CD, Crohn’s disease; IV, intravenous; NR, not reported; RCT, randomised controlled trial; SC, subcutaneous; SD, standard deviation; UC, ulcerative colitis

## Supplementary Figure 1. Forest plots showing the proportion of patients with Crohn’s disease achieving a CDAI-70 response during the induction phase with infliximab (upper plot) or vedolizumab (lower plot)


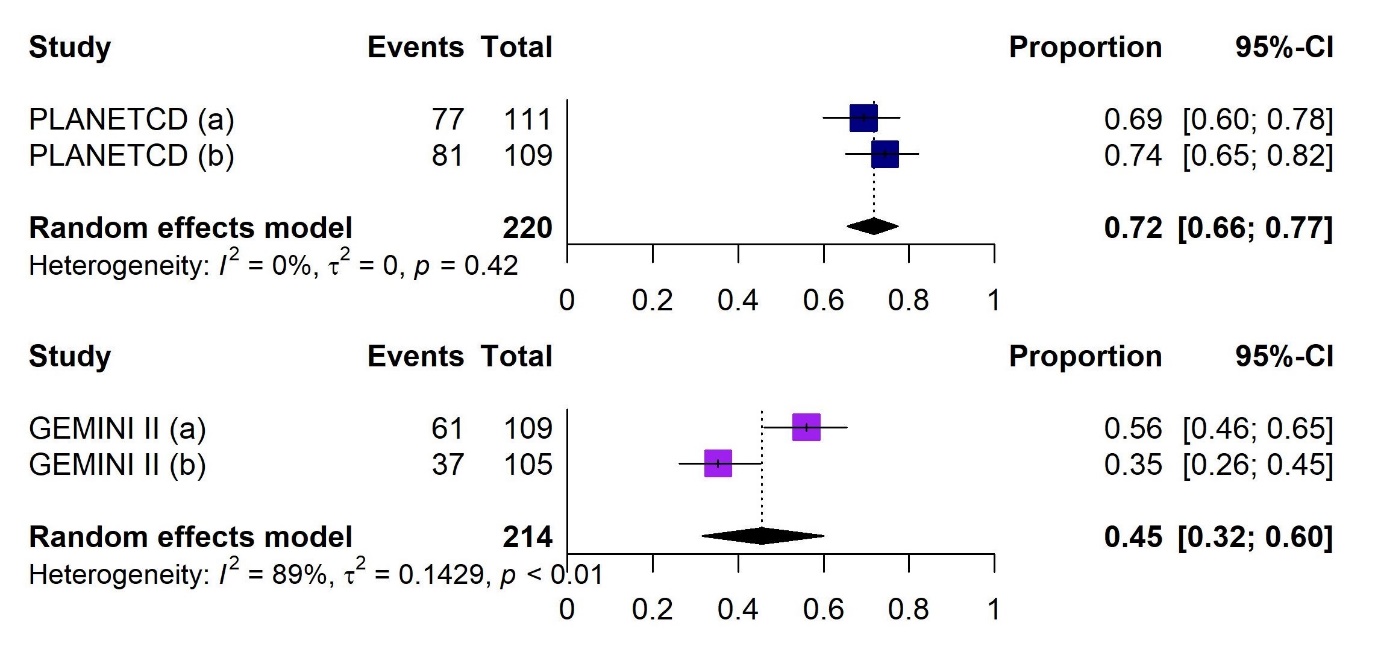


PLANET CD (a): patients with CT-P13 IV only; PLANET CD (b): patients with CT-P13 IV and infliximab IV; GEMINI 2 (a): VDZ before TNFi; GEMINI 2 (b): VDZ after TNFi failure

Abbreviation: CDAI, Crohn’s Disease Activity Index; CI, confidence interval; IV, intravenous; TNFi, tumour necrosis factor-α inhibitor; VDZ, vedolizumab

## Supplementary Figure 2. Forest plots showing the proportion of with Crohn’s disease achieving a CDAI-100 response during the induction phase with infliximab (upper plot) or vedolizumab (lower plot)


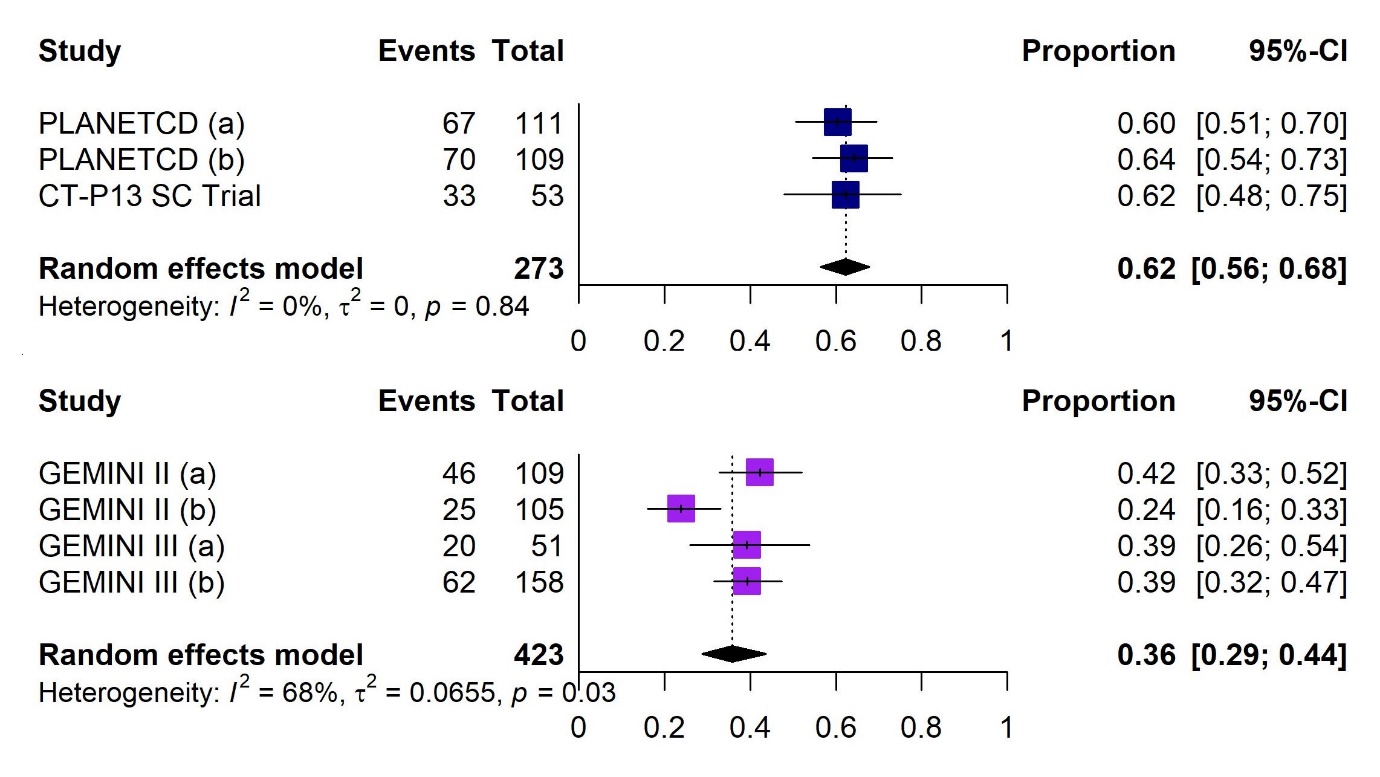


PLANET CD (a): patients with CT-P13 IV only; PLANET CD (b): patients with CT-P13 IV and infliximab IV; GEMINI 2 (a): VDZ before TNFi; GEMINI 2 (b): VDZ after TNFi failure; GEMINI 3 (a): VDZ IV before TNFi; GEMINI 3 (b): VDZ IV after TNFi failure

Abbreviation: CDAI, Crohn’s Disease Activity Index; CI, confidence interval; IV, intravenous; TNFi, tumour necrosis factor-α inhibitor; VDZ, vedolizumab

## Supplementary Figure 3. Forest plots showing the proportion of patients with Crohn’s disease achieving a CDAI-70 response during the maintenance phase with infliximab


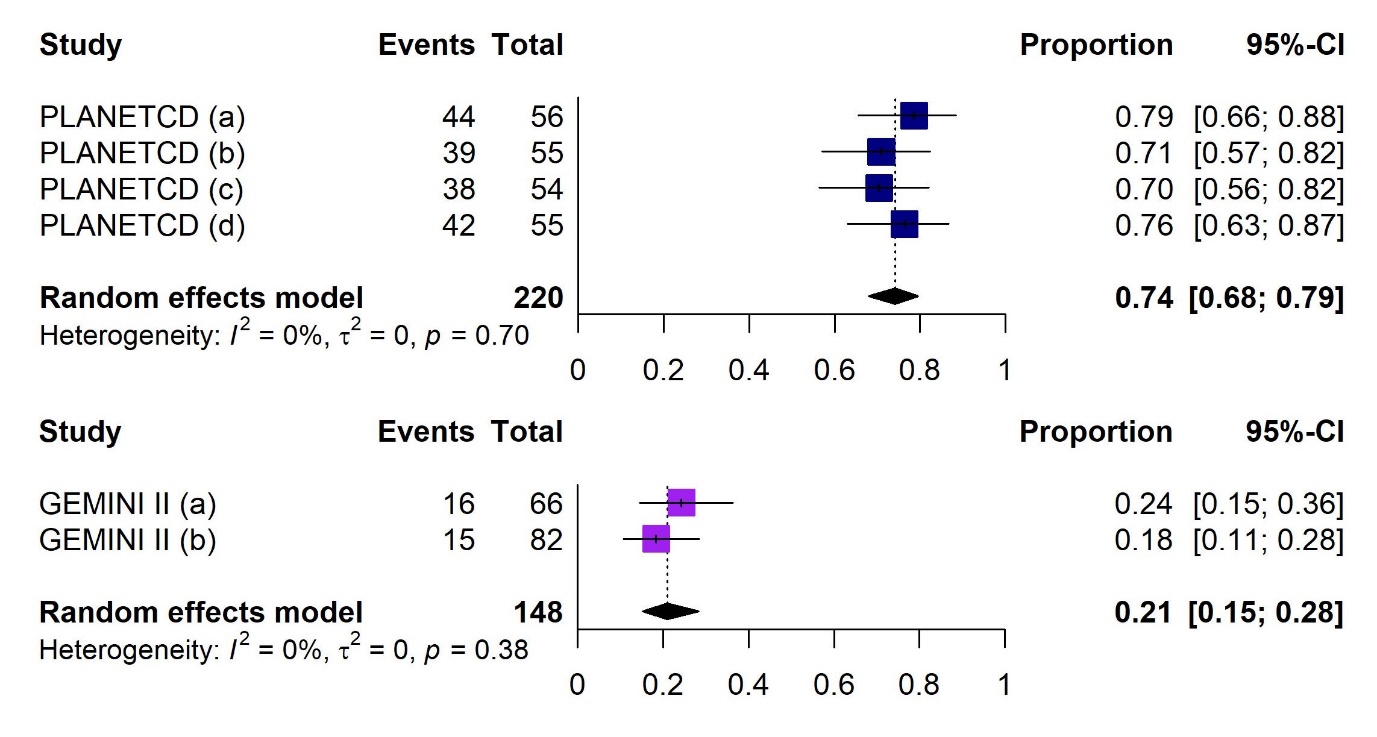


PLANET CD (a): CT-P13 IV only; PLANET CD (b): CT-P13 IV switch to IFX IV; PLANET CD (c): IFX IV only; PLANET CD (d): IFX IV - CT-P13 IV; GEMINI 2 (a): VDZ before TNFi; GEMINI 2 (b): VDZ after TNFi failure

Abbreviation: CI, confidence interval; IFX, infliximab; IV, intravenous; TNFi, tumour necrosis factor-α inhibitor; VDZ, vedolizumab

## Supplementary Figure 4. Forest plots showing the proportion of patients with Crohn’s disease achieving a CDAI-100 response during the maintenance phase with infliximab (upper plot) or vedolizumab (lower plot)


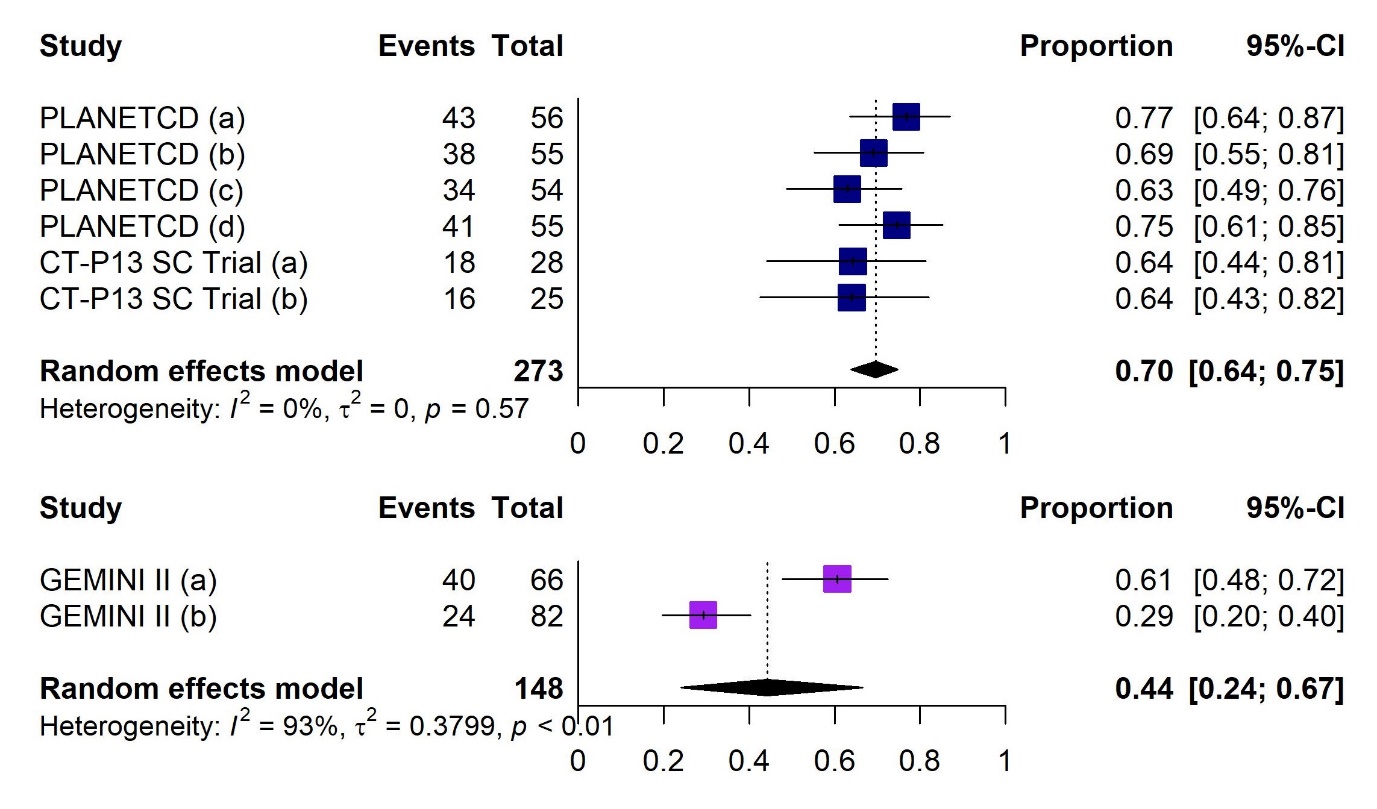


PLANET CD (a): CT-P13 IV only; PLANET CD (b): CT-P13 IV switch to IFX IV; PLANET CD (c): IFX IV only; PLANET CD (d): IFX IV switch to CT-P13 IV; CT-P13 SC trial (a): CT-P13 SC only; CT-P13 SC trial (b): CT-P13 IV switch to CT-P13 SC; GEMINI 2 (a): VDZ before TNFi; GEMINI 2 (b): VDZ after TNFi failure

Abbreviation: CDAI, Crohn’s Disease Activity Index; CI, confidence interval; IFX, infliximab; IV, intravenous; SC, subcutaneous; TNFi, tumour necrosis factor-α inhibitor; VDZ, vedolizumab

## Supplementary Figure 5. Forest plots showing the proportion of patients with Crohn’s disease experiencing any adverse event with infliximab (upper plot) or vedolizumab (lower plot)


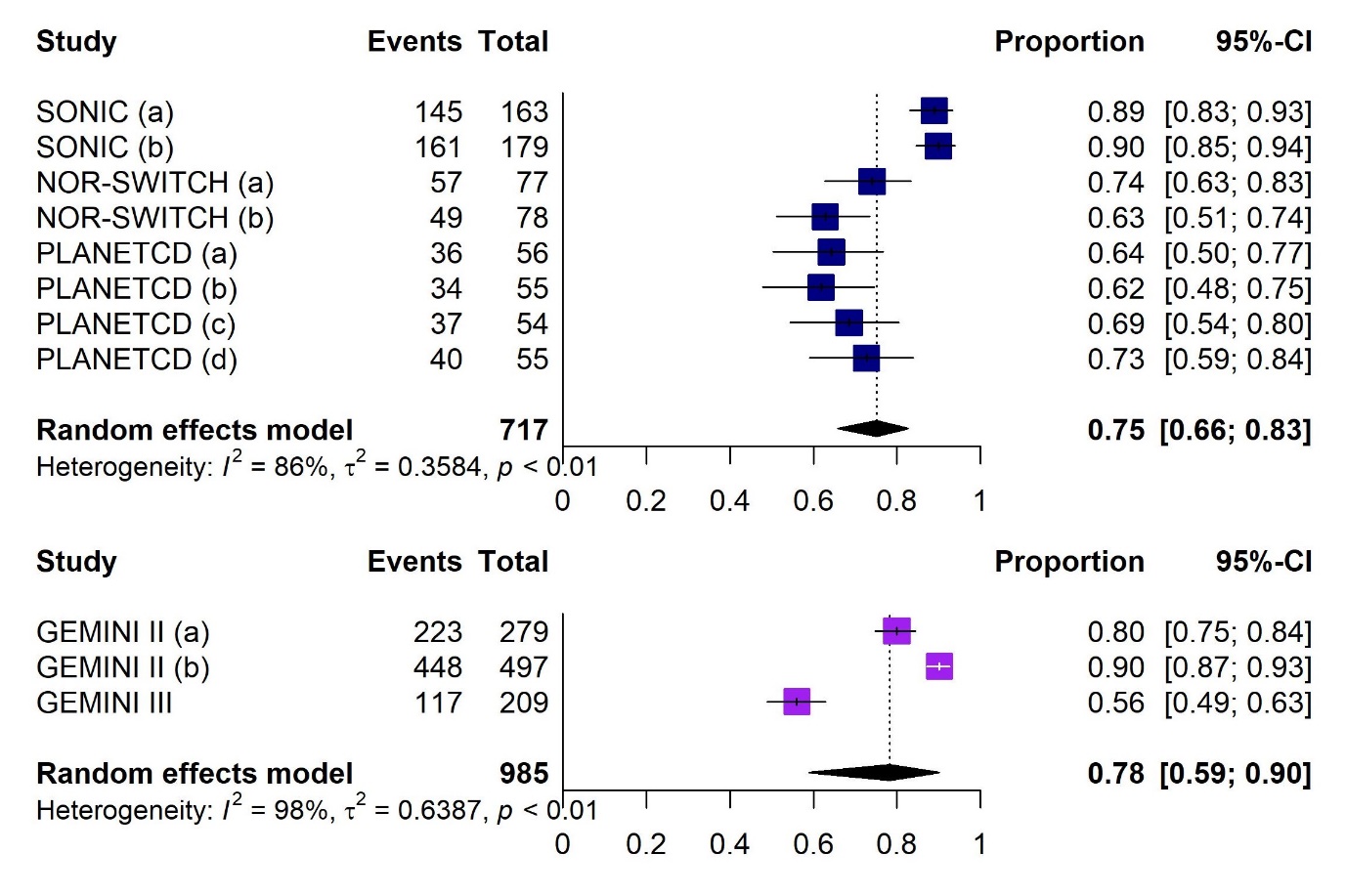


SONIC (a): IFX IV (corticosteroid free); SONIC (b): combination therapy; NOR-SWITCH (a): IFX (CT-P13); NOR-SWITCH (b): IFX (other); PLANET CD (a): CT-P13 IV only; PLANET CD (b): CT-P13 IV switch to IFX IV; PLANET CD (c): IFX IV only; PLANET CD (d): IFX IV switch to CT-P13 IV; CT-P13 SC trial (a): CT-P13 SC only; CT-P13 SC trial (b): CT-P13 IV switch to CT-P13 SC; GEMINI 2 (a): VDZ before TNFi; GEMINI 2 (b): VDZ after TNFi failure

Abbreviation: CI, confidence interval; IFX, infliximab; IV, intravenous; SC, subcutaneous; TNFi, tumour necrosis factor-α inhibitor; VDZ, vedolizumab

## Supplementary Figure 6. Forest plots showing the proportion of patients with Crohn’s disease experiencing any serious adverse event with infliximab (upper plot) or vedolizumab (lower plot)


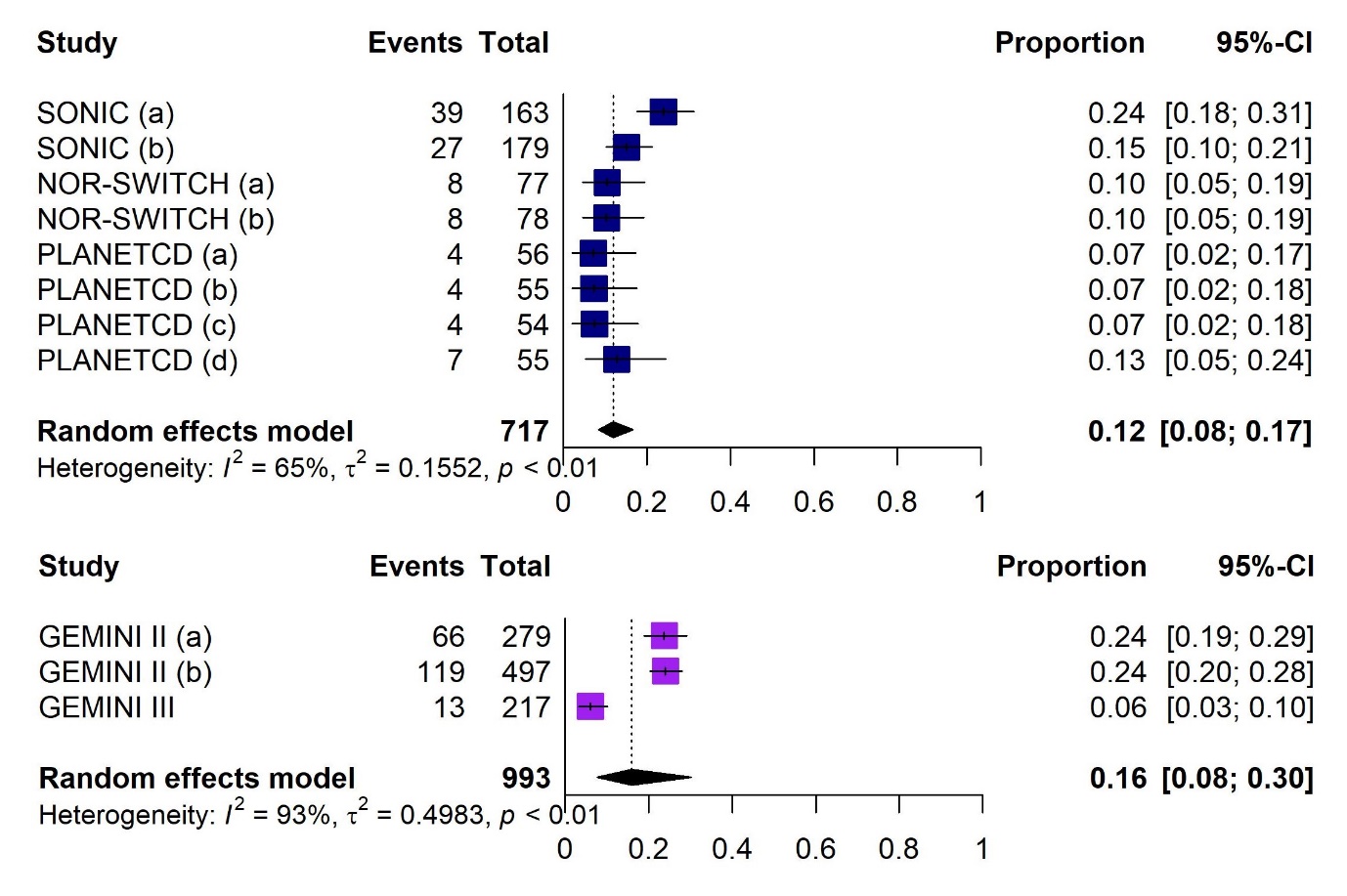


SONIC (a): IFX IV (corticosteroid free); SONIC (b): combination therapy; NOR-SWITCH (a): IFX (CT-P13); NOR-SWITCH (b): IFX (other); PLANET CD (a): CT-P13 IV only; PLANET CD (b): CT-P13 IV switch to IFX IV; PLANET CD (c): IFX IV only; PLANET CD (d): IFX IV switch to CT-P13 IV; GEMINI 2 (a): VDZ before TNFi; GEMINI 2 (b): VDZ after TNFi failure

Abbreviation: CI, confidence interval; IFX, infliximab; IV, intravenous; TNFi, tumour necrosis factor-α inhibitor; VDZ, vedolizumab

## Supplementary Figure 7. Forest plots showing the proportion of patients with Crohn’s disease experiencing any infection with infliximab (upper plot) or vedolizumab (lower plot)


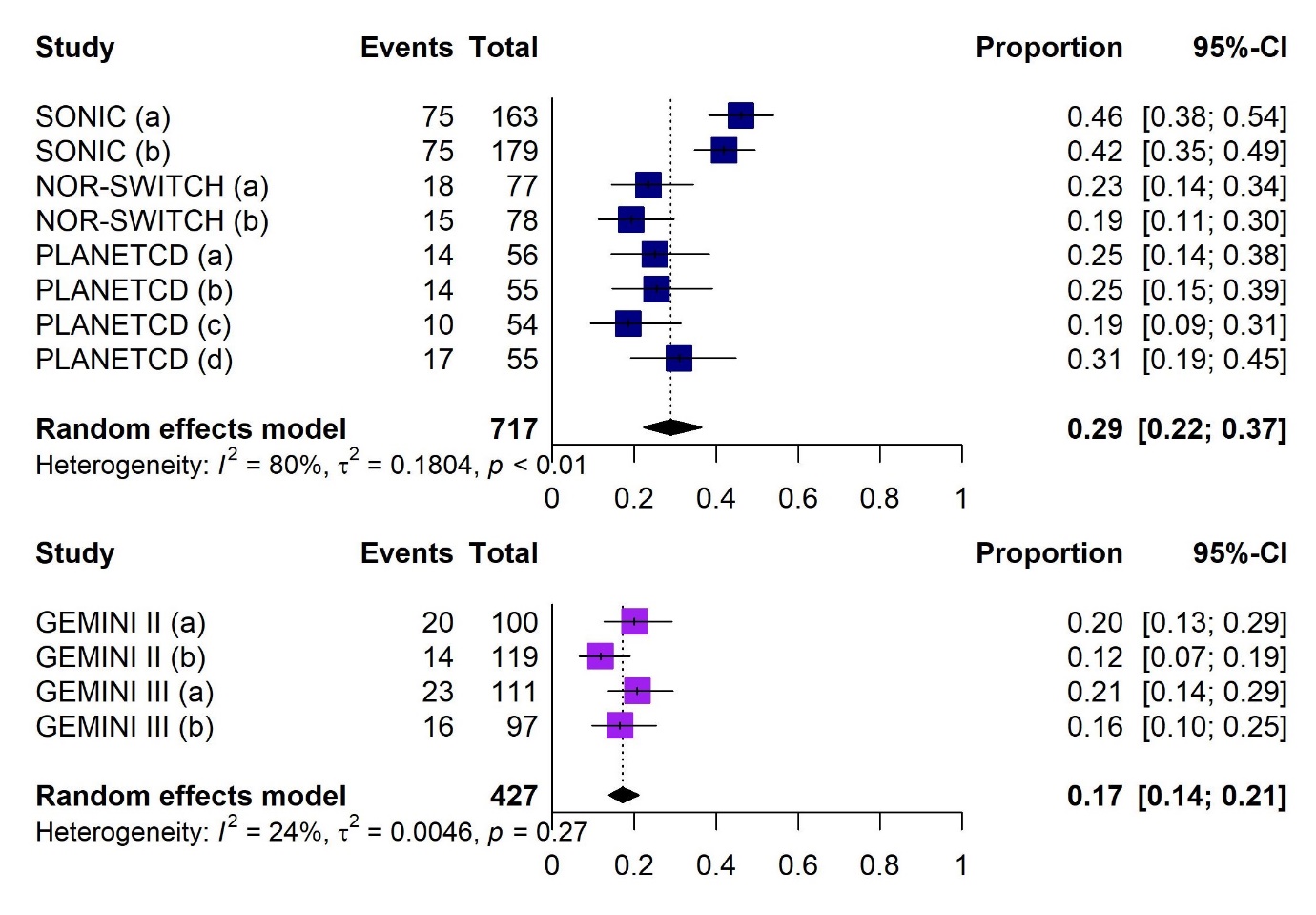


SONIC (a): IFX IV (corticosteroid free); SONIC (b): combination therapy; NOR-SWITCH (a): IFX (CT-P13); NOR-SWITCH (b): IFX (other); PLANET CD (a): CT-P13 IV only; PLANET CD (b): CT-P13 IV switch to IFX IV; PLANET CD (c): IFX IV only; PLANET CD (d): IFX IV switch to CT-P13 IV; GEMINI 2 (a): VDZ with corticosteroids; GEMINI 2 (b): VDZ without corticosteroids; GEMINI 3 (a): VDZ with corticosteroids; GEMINI 3 (b): VDZ without corticosteroids

Abbreviation: CI, confidence interval; IFX, infliximab; IV, intravenous; VDZ, vedolizumab

## Supplementary Figure 8. Forest plots showing the proportion of patients with Crohn’s disease experiencing any serious infection with infliximab (upper plot) or vedolizumab (lower plot)


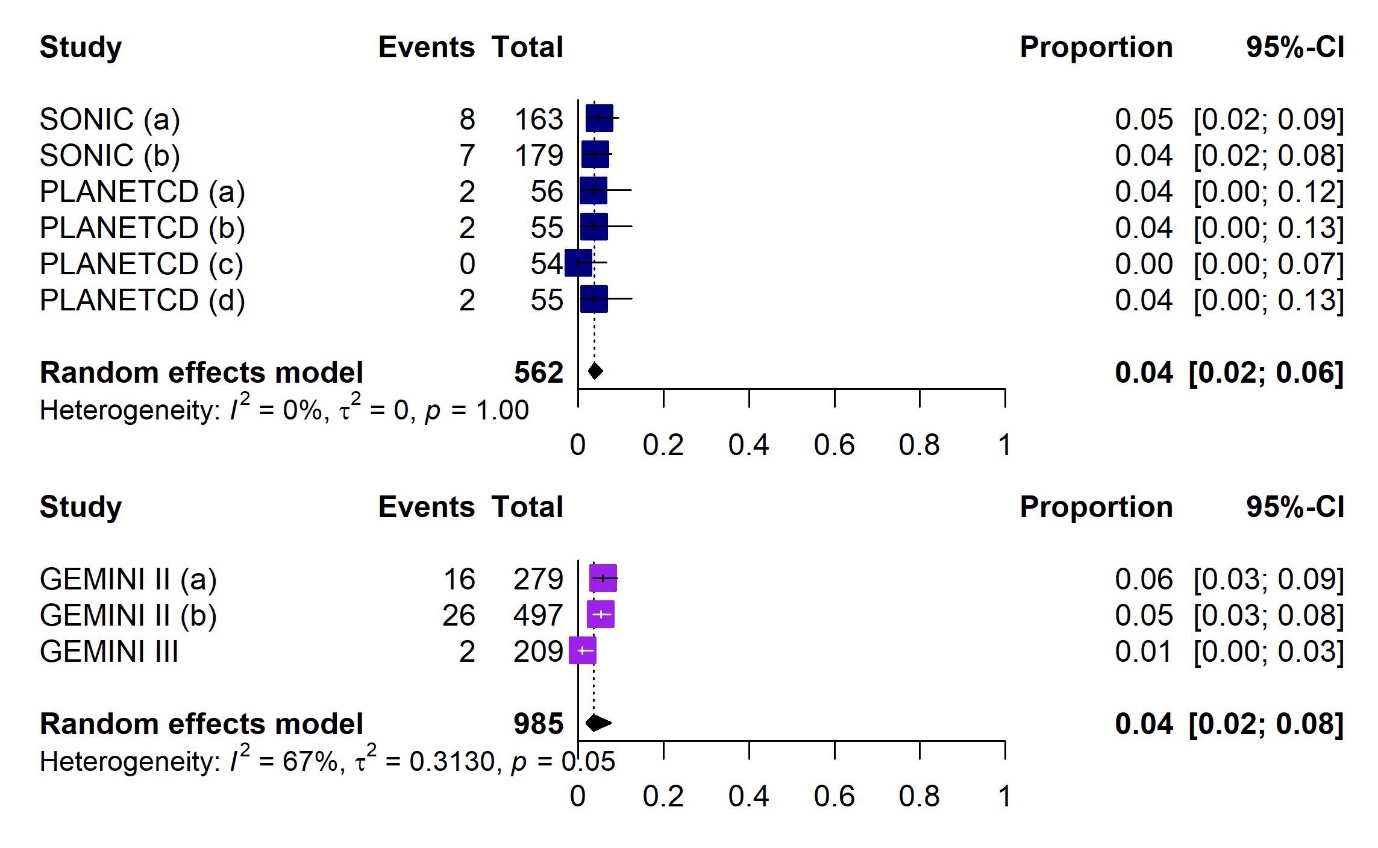


SONIC (a): IFX IV (corticosteroid free); SONIC (b): combination therapy; PLANET CD (a): CT-P13 IV only; PLANET CD (b): CT-P13 IV switch to IFX IV; PLANET CD (c): IFX IV only; PLANET CD (d): IFX IV switch to CT-P13 IV; GEMINI 2 (a): VDZ before TNFi; GEMINI 2 (b): VDZ after TNFi failure

Abbreviation: CI, confidence interval; IFX, infliximab; IV, intravenous; TNFi, tumour necrosis factor-α inhibitor; VDZ, vedolizumab

## Supplementary Figure 9. Forest plots showing the proportion of patients with Crohn’s disease who discontinued due to adverse events in the infliximab (upper plot) or vedolizumab (lower plot) treatment arms


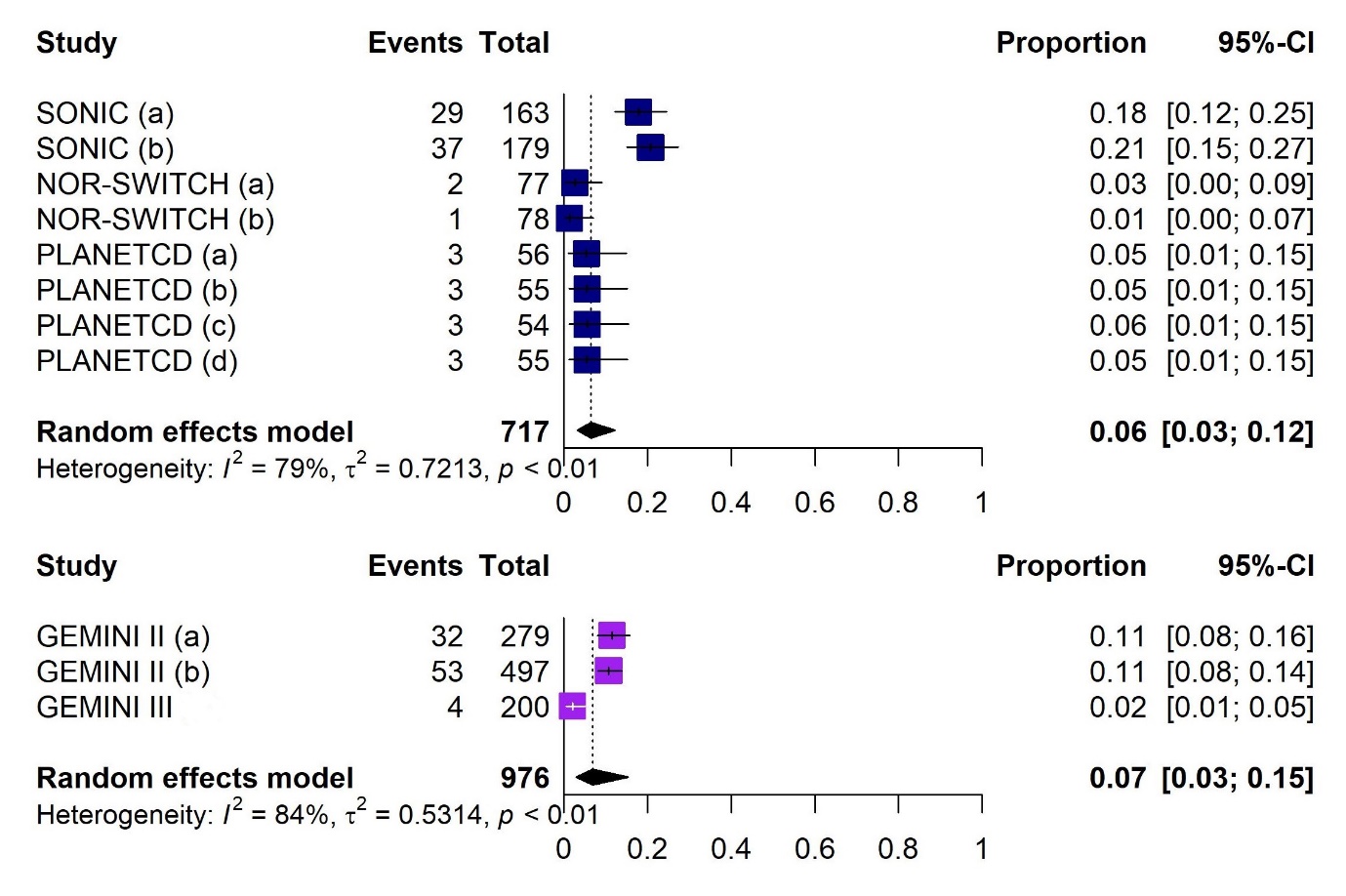


SONIC (a): IFX IV (corticosteroid free); SONIC (b): combination therapy; NOR-SWITCH (a): IFX (CT-P13); NOR-SWITCH (b): IFX (other); PLANET CD (a): CT-P13 IV only; PLANET CD (b): CT-P13 IV switch to IFX IV; PLANET CD (c): IFX IV only; PLANET CD (d): IFX IV switch to CT-P13 IV; GEMINI 2 (a): VDZ with corticosteroids; GEMINI 2 (b): VDZ without corticosteroids

Abbreviation: CI, confidence interval; IFX, infliximab; IV, intravenous; VDZ, vedolizumab

## Supplementary Figure 10. Forest plots showing the proportion of patients with Crohn’s disease who discontinued due to lack of efficacy in the infliximab treatment arm

**
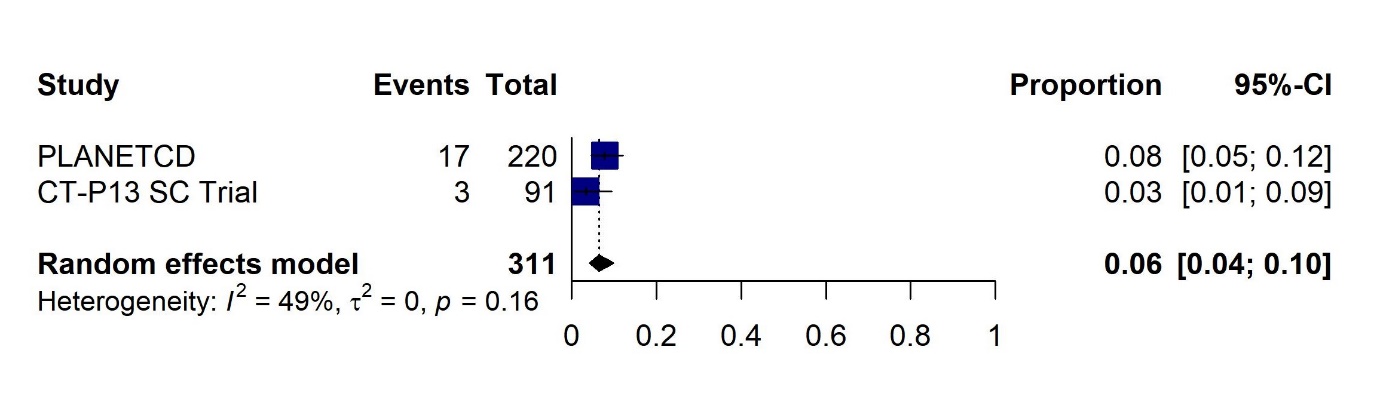
**

Abbreviation: CI, confidence interval; SC, subcutaneous

## Supplementary Figure 11. Forest plots showing the proportion of patients with ulcerative colitis achieving a clinical response during the induction phase with infliximab (upper plot) or vedolizumab (lower plot)


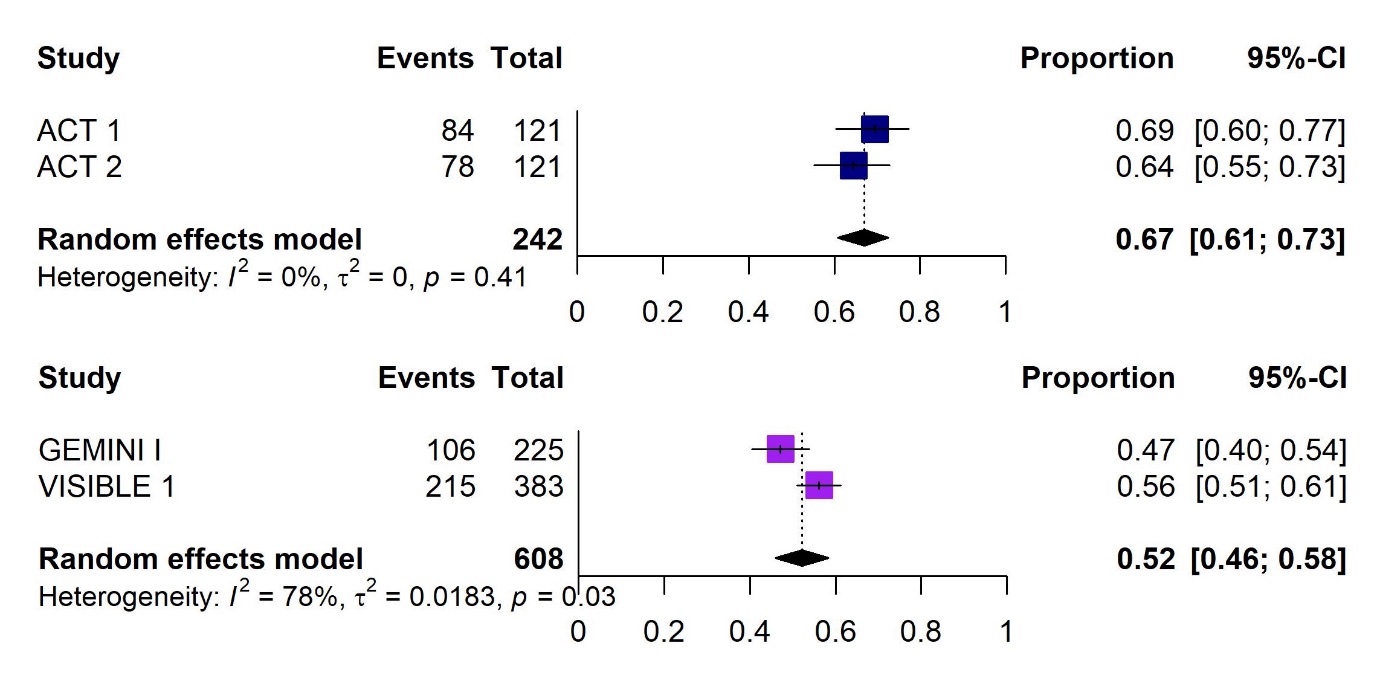


Abbreviation: CI, confidence interval

## Supplementary Figure 12. Forest plot showing the proportion of patients with ulcerative colitis achieving mucosal healing during the induction phase with infliximab


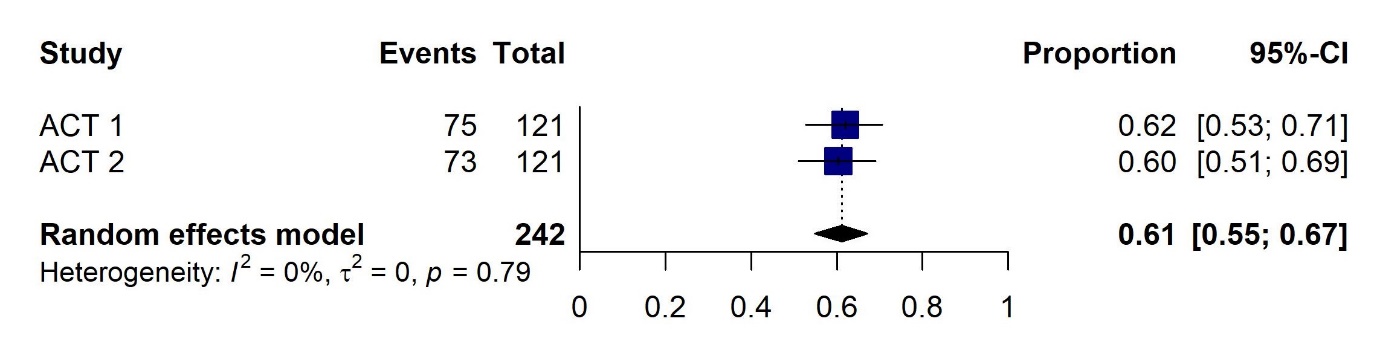


Abbreviation: CI, confidence interval

## Supplementary Figure 13. Forest plots showing the proportion of patients with ulcerative colitis achieving a clinical response during the maintenance phase with infliximab (upper plot) or vedolizumab (lower plot)


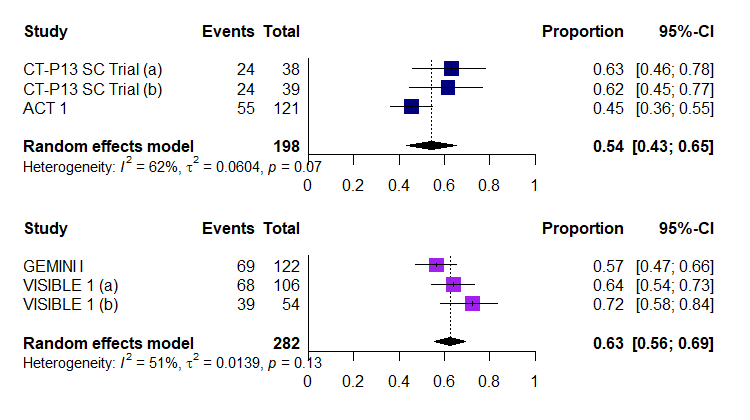


CT-P13 SC trial (a): CT-P13 SC only; CT-P13 SC trial (b): CT-P13 IV switch to CT-P13 SC; VISIBLE 1 (a): VDZ SC; VISIBLE 1 (b): VDZ IV

Abbreviation: CI, confidence interval; IV, intravenous; SC, subcutaneous

## Supplementary Figure 14. Forest plots showing the proportion of patients with ulcerative colitis achieving mucosal healing during the maintenance phase with infliximab (upper plot) or vedolizumab (lower plot)


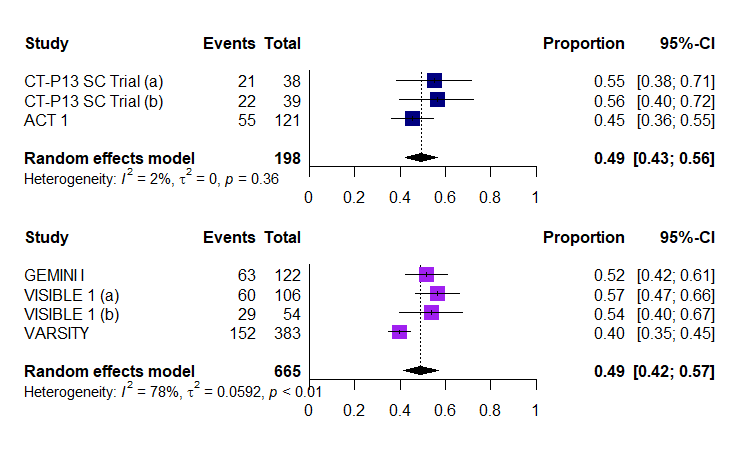


CT-P13 SC trial (a): CT-P13 SC only; CT-P13 SC trial (b): CT-P13 IV switch to CT-P13 SC; VISIBLE 1 (a): VDZ SC; VISIBLE 1 (b): VDZ IV

Abbreviation: CI, confidence interval; IV, intravenous; SC, subcutaneous

## Supplementary Figure 15. Forest plots showing the proportion of patients with ulcerative colitis experiencing any adverse event with infliximab (upper plot) or vedolizumab (lower plot)


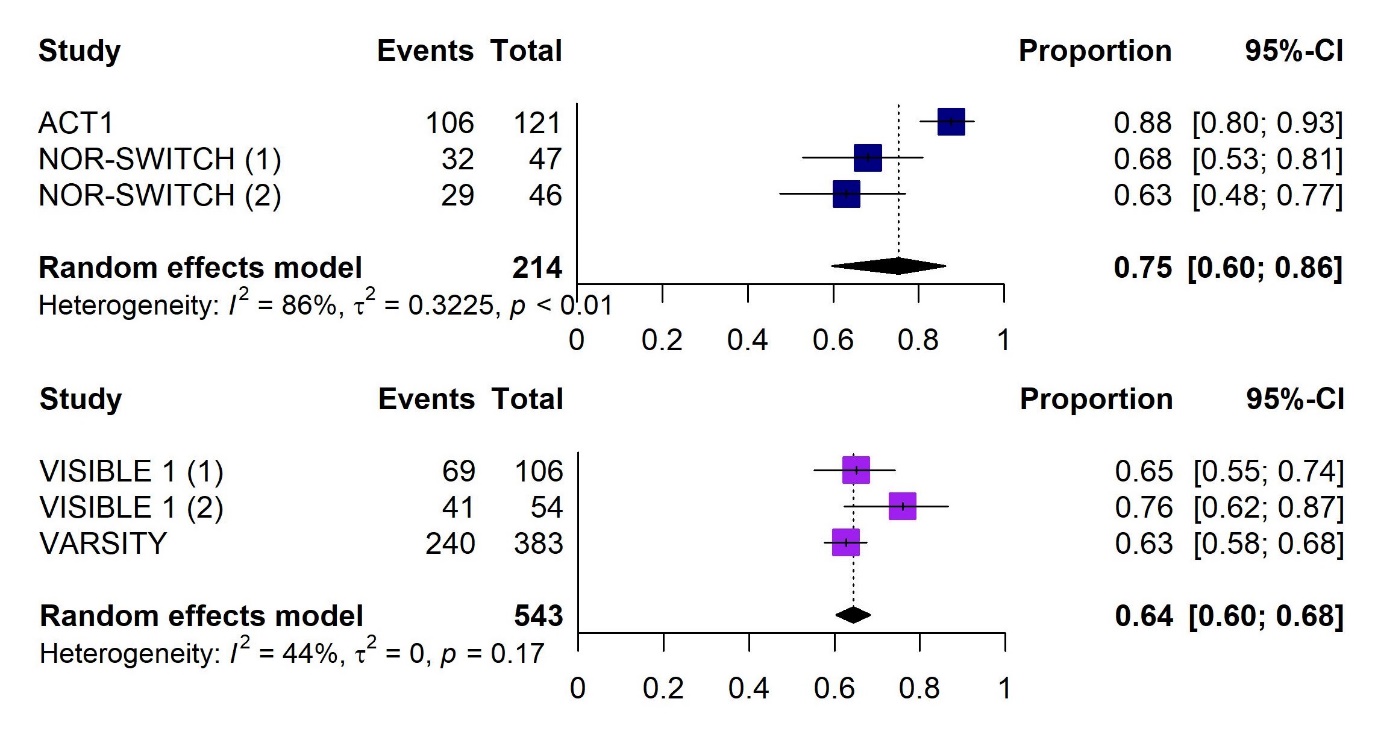


NOR-SWITCH (a): originator IFX IV; NOR-SWITCH (b): CT-P13; VISIBLE 1 (a): VDZ SC; VISIBLE 1 (b): VDZ IV

Abbreviation: CI, confidence interval; IFX, infliximab; IV, intravenous; SC, subcutaneous

## Supplementary Figure 16. Forest plots showing the proportion of patients with ulcerative colitis experiencing any serious adverse event with infliximab (upper plot) or vedolizumab (lower plot)


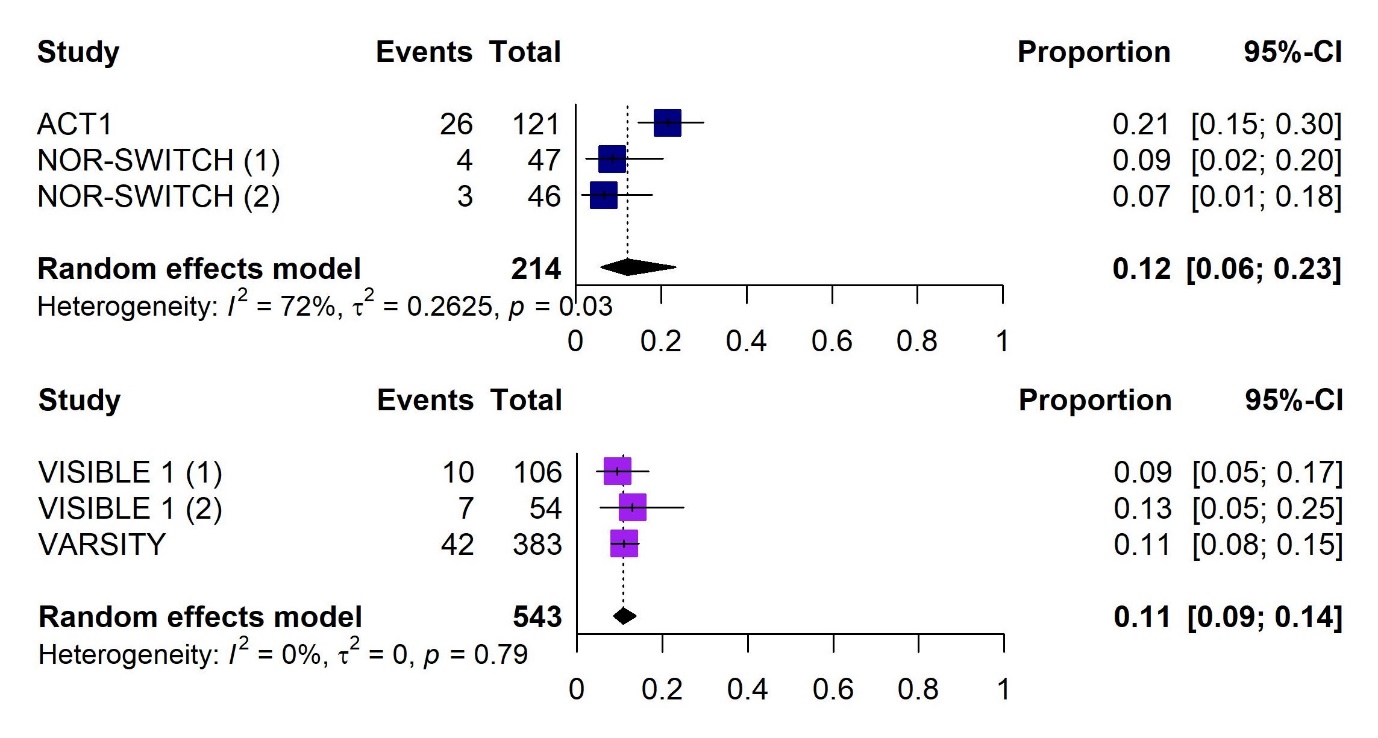


NOR-SWITCH (a): originator IFX IV; NOR-SWITCH (b): CT-P13; VISIBLE 1 (a): VDZ SC; VISIBLE 1 (b): VDZ IV

Abbreviation: CI, confidence interval; IFX, infliximab; IV, intravenous

## Supplementary Figure 17. Forest plots showing the proportion of patients with ulcerative colitis experiencing any infection with infliximab (upper plot) or vedolizumab (lower plot)


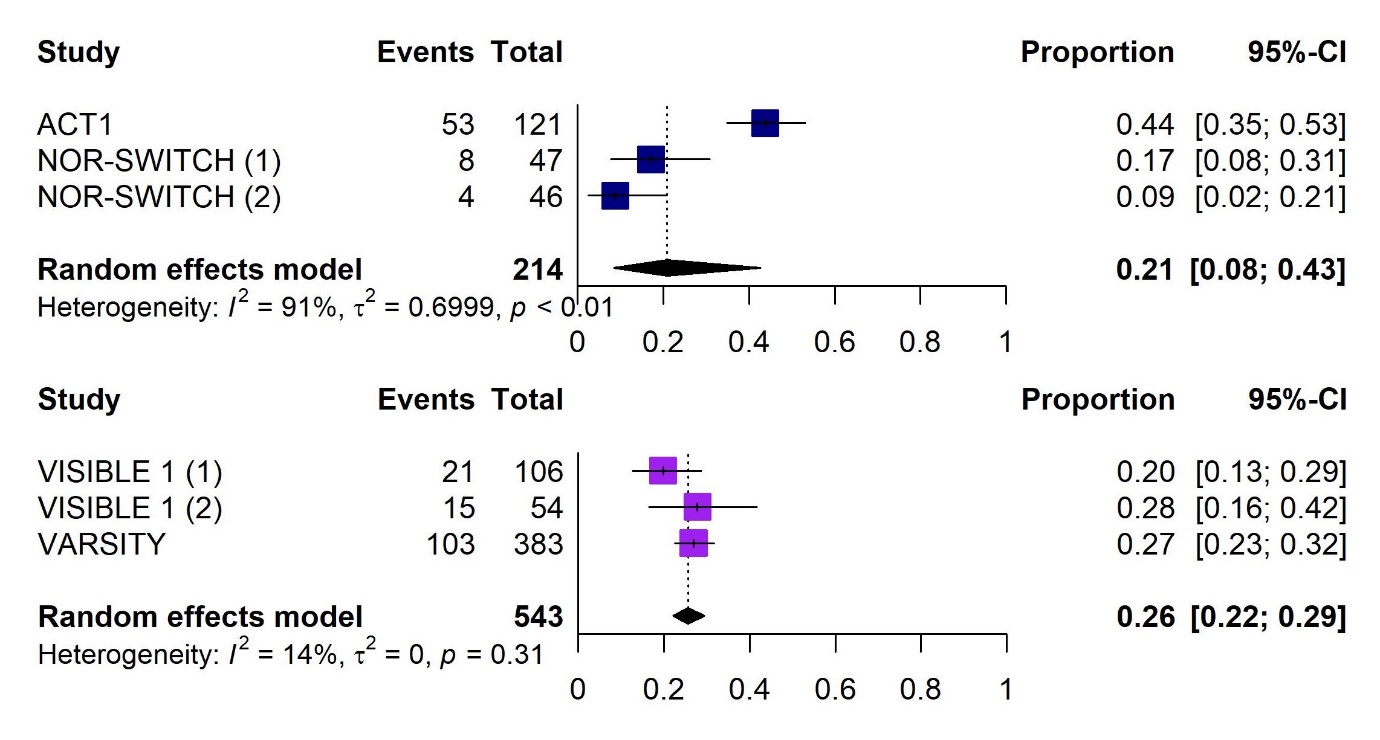


NOR-SWITCH (a): originator IFX IV; NOR-SWITCH (b): CT-P13; VISIBLE 1 (a): VDZ SC; VISIBLE 1 (b): VDZ IV

Abbreviation: CI, confidence interval; IFX, infliximab; IV, intravenous

## Supplementary Figure 18. Forest plots showing the proportion of patients with ulcerative colitis experiencing any serious infection with infliximab (upper plot) or vedolizumab (lower plot)


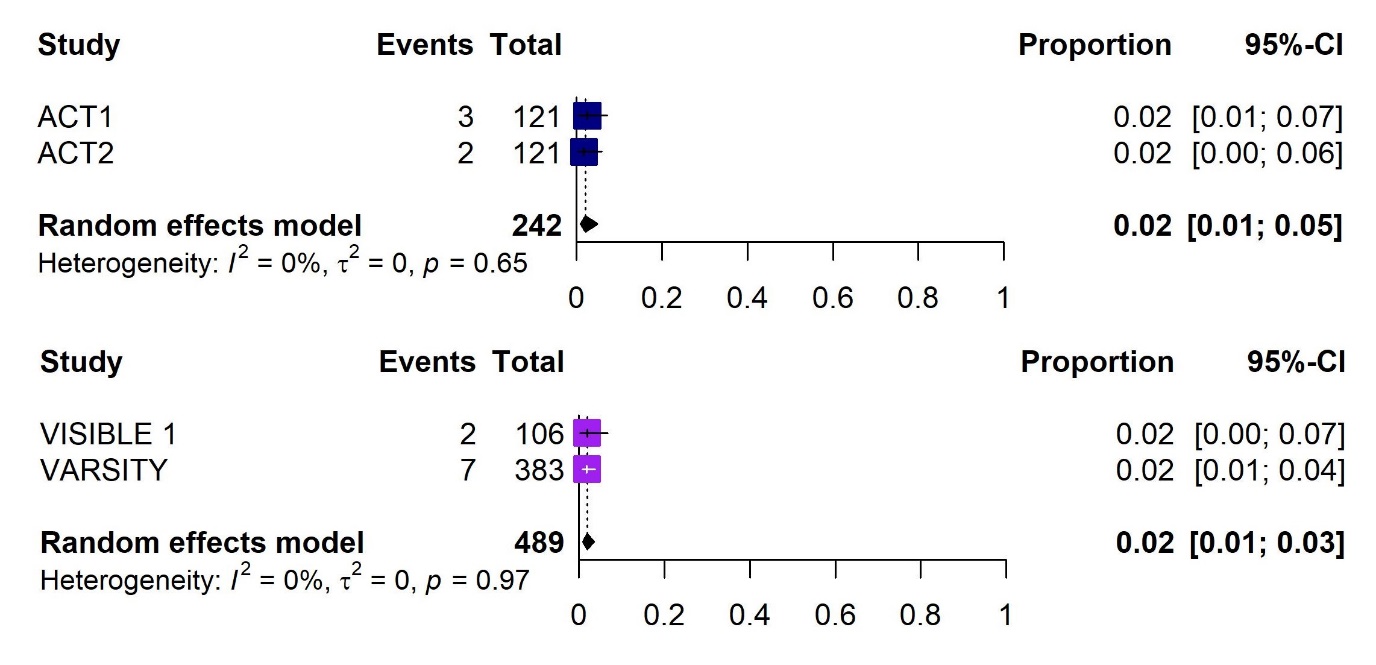


Abbreviation: CI, confidence interval

## Supplementary Figure 19. Forest plots showing the proportion of patients with ulcerative colitis who discontinued due to adverse events with infliximab (upper plot) or vedolizumab (lower plot)


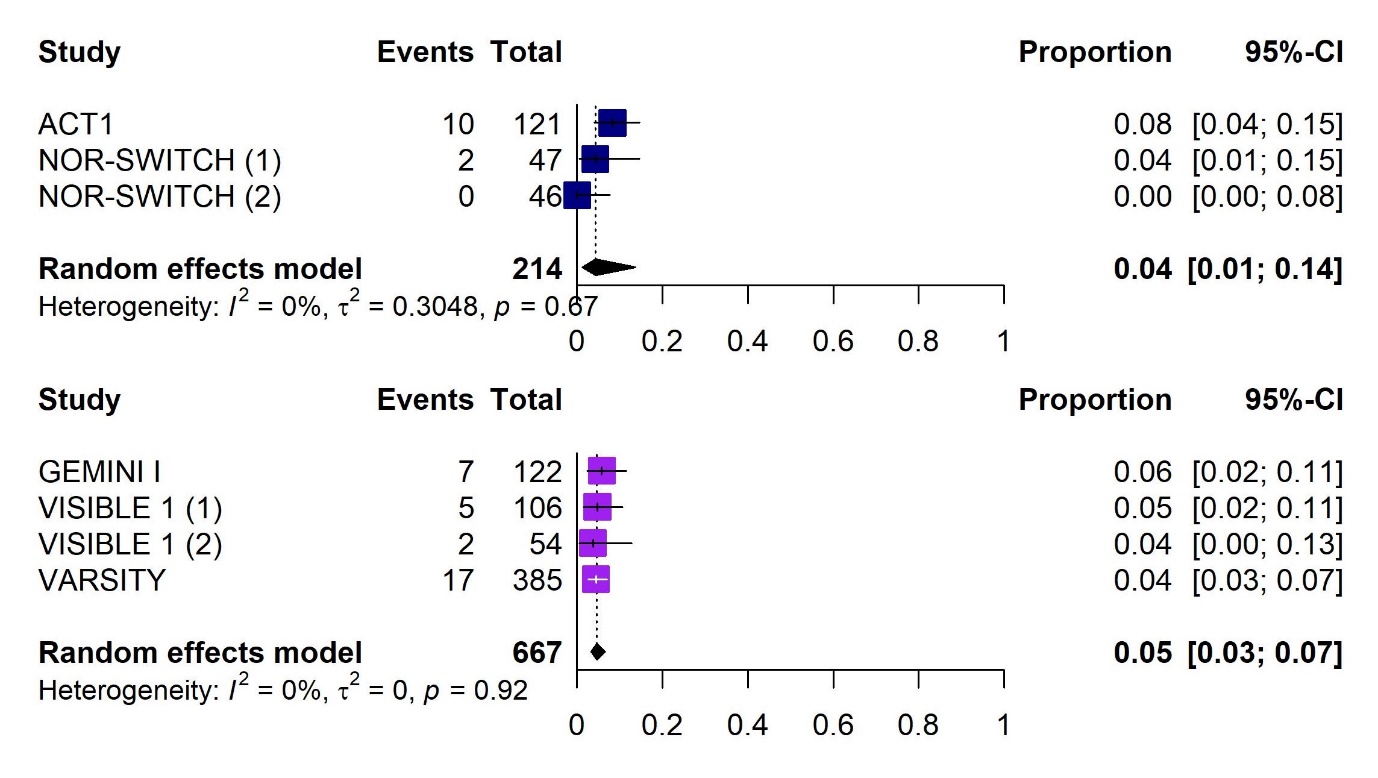


NOR-SWITCH (a): originator IFX IV; NOR-SWITCH (b): CT-P13; VISIBLE 1 (a): VDZ SC; VISIBLE 1 (b): VDZ IV

Abbreviation: CI, confidence interval; IFX, infliximab; IV, intravenous

## Supplementary Figure 20. Forest plots showing the proportion of patients with ulcerative colitis who discontinued due to lack of efficacy with vedolizumab

**
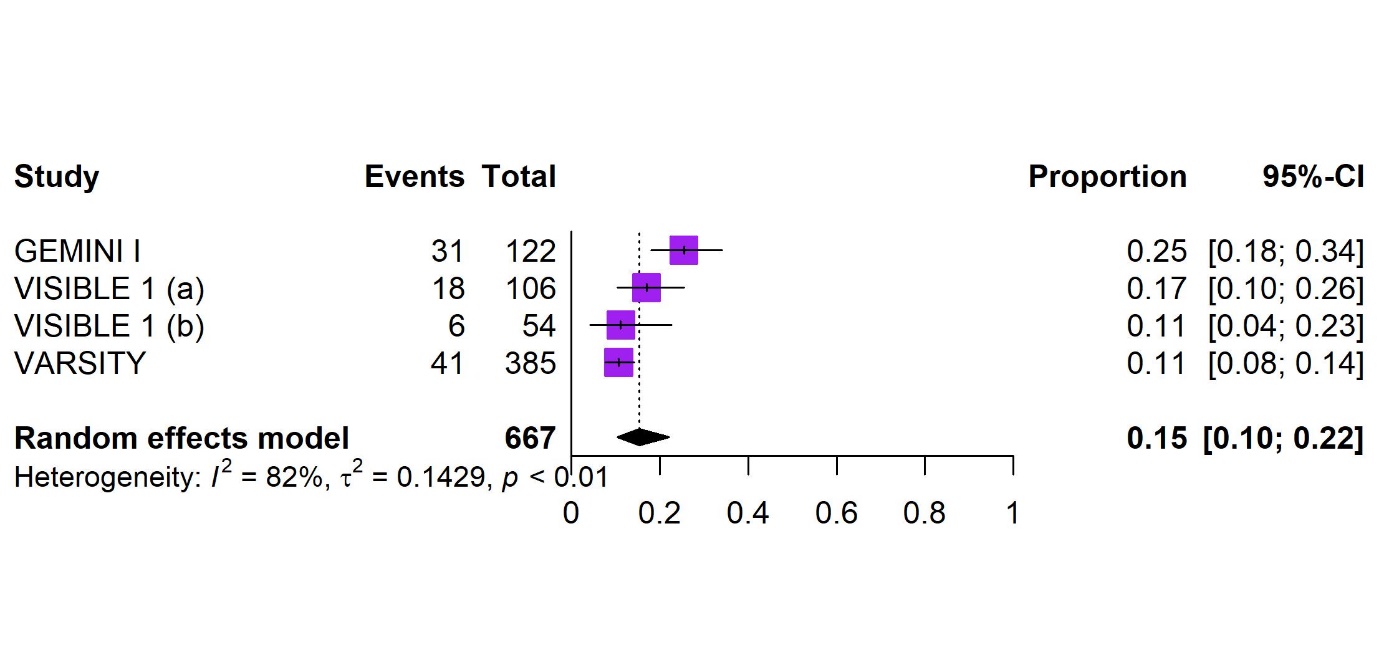
**

VISIBLE 1 (a): VDZ SC; VISIBLE 1 (b): VDZ IV

Abbreviation: CI, confidence interval

# References

- 1. Colombel JF, Sandborn WJ, Reinisch W, et al. Infliximab, azathioprine, or combination therapy for Crohn's disease *N Engl J Med*. 2010;362:1383–1395.
- 2. Ye BD, Pesegova M, Alexeeva O, et al. Efficacy and safety of biosimilar CT-P13 compared with originator infliximab in patients with active Crohn's disease: an international, randomised, double-blind, phase 3 non-inferiority study *Lancet*. 2019;393:1699–1707.
- 3. Jorgensen KK, Goll GL, Sexton J, et al. Efficacy and safety of CT-P13 in inflammatory bowel disease after switching from originator infliximab: Exploratory analyses from the NOR-SWITCH main and extension trials *BioDrugs*. 2020;34:681–694.
- 4. Jørgensen KK, Olsen IC, Goll GL, et al. Switching from originator infliximab to biosimilar CT-P13 compared with maintained treatment with originator infliximab (NOR-SWITCH): a 52-week, randomised, double-blind, non-inferiority trial *Lancet*. 2017;389:2304–2316.
- 5. Schreiber S, Ben-Horin S, Leszczyszyn J, et al. Randomized controlled trial: Subcutaneous vs intravenous infliximab CT-P13 maintenance in inflammatory bowel disease *Gastroenterology*. 2021;160:2340–2353.
- 6. Rutgeerts P, Sandborn WJ, Feagan BG, et al. Infliximab for induction and maintenance therapy for ulcerative colitis *N Engl J Med*. 2005;353:246–276.
- 7. Sandborn WJ, Feagan BG, Rutgeerts P, et al. Vedolizumab as induction and maintenance therapy for Crohn's disease *N Engl J Med*. 2013;369:711–721.
- 8. Sands BE, Feagan BG, Rutgeerts P, et al. Effects of vedolizumab induction therapy for patients with Crohn's disease in whom tumor necrosis factor antagonist treatment failed *Gastroenterology*. 2014;147:618–627.e613.
- 9. Feagan BG, Rutgeerts P, Sands BE, et al. Vedolizumab as induction and maintenance therapy for ulcerative colitis *N Engl J Med*. 2013;369:699–710.
- 10. Sands BE, Peyrin-Biroulet L, Loftus EV, Jr., et al. Vedolizumab versus adalimumab for moderate-to-severe ulcerative colitis *N Engl J Med*. 2019;381:1215–1226.
- 11. Sandborn WJ, Baert F, Danese S, et al. Efficacy and safety of vedolizumab subcutaneous formulation in a randomized trial of patients with ulcerative colitis *Gastroenterology*. 2020;158:562–572.e512.
